# Phosphokinase Antibody Arrays on Dendron-Coated Surface

**DOI:** 10.1371/journal.pone.0096456

**Published:** 2014-05-06

**Authors:** Ju-Won Kwak, Hyobin Jeong, Sun-Ho Han, Youngkyu Kim, Sung Min Son, Inhee Mook-Jung, Daehee Hwang, Joon Won Park

**Affiliations:** 1 Department of Chemistry, POSTECH, Pohang, Republic of Korea; 2 School of Interdisciplinary Bioscience and Bioengineering, POSTECH, Pohang, Republic of Korea; 3 Department of Biochemistry and Biomedical Sciences, Seoul National University, Seoul, Republic of Korea; 4 Center for Plant Aging Research, Institute for Basic Science, DGIST, Daegu, Republic of Korea; Heidelberg University, Germany

## Abstract

Monitoring protein phosphorylation at the cellular level is important to understand the intracellular signaling. Among the phosphoproteomics methods, phosphokinase antibody arrays have emerged as preferred tools to measure well-characterized phosphorylation in the intracellular signaling. Here, we present a dendron-coated phosphokinase antibody array (DPA) in which the antibodies are immobilized on a dendron-coated glass slide. Self-assembly of conically shaped dendrons well-controlled in size and structure resulted in precisely controlled lateral spacing between the immobilized phosphosite-specific antibodies, leading to minimized steric hindrance and improved antigen-antibody binding kinetics. These features increased sensitivity, selectivity, and reproducibility in measured amounts of protein phosphorylation. To demonstrate the utility of the DPA, we generated the phosphorylation profiles of brain tissue samples obtained from Alzheimer's disease (AD) model mice. The analysis of the profiles revealed signaling pathways deregulated during the course of AD progression.

## Introduction

Profiling protein phosphorylation at the cellular level is essential to understand the intracellular signaling upon external and internal stimulations. Non-targeted mass spectrometry (MS)-based phosphoproteomic approaches have been used for profiling protein phosphorylation. These methods involve isolation of phosphorylated peptides using affinity chromatography methods [1], [2] followed by liquid chromatography (LC)-MS/MS analysis of the isolated phosphorylated peptides. However, the isolation of phosphorylated peptides and LC-MS/MS analysis, which employs the data-dependent acquisition, introduce biases toward detection of abundant phosphorylated peptides with no guarantee for detecting well-characterized phosphosites [3]. To partially resolve this issue, targeted multiple reaction monitoring (MRM)-based phosphoproteomic approaches have been developed to monitor a number of selected phosphorylated peptides [4].

Despite the capability of the MS-based methods in discovering novel phosphosites, phosphokinase arrays have emerged as preferred tools to measure well-characterized phosphosites of functional significance using the phosphosite-specific antibodies. These arrays can be categorized into antibody arrays [5], [6] and reverse-phase protein lysate arrays [7]–[9]. Several phosphokinase antibody arrays have been developed (**Table S1**), including Phospho Explorer Array (Full Moon BioSystems), Signal Transduction AntibodyArray (Hypromatrix), Kinex Antibody Microarray (Kinexus Bioinformatics), Human Phospho-Kinase Antibody Array (R&D system), and Panorama Antibody Array XP725 (Sigma-Aldrich). Compared to the MS-based methods, these array-based methods require small amounts of the samples (**Table S1**). A major limiting factor for development of antibody arrays is the availability of high-quality phosphosite-specific antibodies. Recently, more and more phosphosite-specific antibodies have been developed. The above commercial phosphokinase antibody arrays include 27 to 675 phosphosite-specific antibodies (**Table S1**). These arrays have been used to analyze alterations of phosphoproteomes at various cellular conditions. For example, Xiao et al. [10] identified a β-arrestin-mediated signaling network in HEK293 cells using Human Phospho-Kinase Array. Also, Allaeys et al. [11] found that monosodium urate microcrystals activate phagocytosis and NLRP3-dependent autophagy in human osteoblast cells by decreasing phosphorylation of TOR using the phosphokinase arrays from R&D system. Moreover, El-Haibi et al. [12] identified downstream signaling networks of Cxcl13 and Cxcr5 in PC3 prostate cancer cells using the phosphokinase arrays from Full Moon Biosystems.

Due to the low abundance of phosphorylated proteins [13], an appropriate surface coating on the surface and immobilization of phosphosite-specific antibodies on the surface are critical to reduce steric hindrance and enhance antigen-antibody binding kinetics [14]–[16]. For example, Cretich et al. [15] showed that a 3-dimensional surface coated with a copolymer of N,N-dimethylacrylamide, N,N-acryloyloxysuccinimide, and [3-(methacryloyl-oxy)propyl]trimethoxysilyl enhanced accessibility of antigens to antibodies on the surface by reducing steric hindrance. Also, Dai et al. [16] found that multilayer poly acrylic acid (PAAs) coated on a microporous surface led to covalent immobilization of antibodies after activation of the free –COOH groups of PAAs, which enhanced the detection sensitivity by two orders of magnitude. Most commercial phosphokinase arrays immobilize phosphosite-specific antibodies on nitrocellulose membranes or polymer-coated glass slides (**Table S1**). Here, we present a dendron-coated phosphokinase antibody array (DPA). In the DPA, a conically shaped dendron with a uniform molecular weight and size and well-defined structure were self-assembled on the surface, and phosphosite-specific antibodies were then conjugated on the apexes of the immobilized dendrons through the specific covalent bond. Therefore, the spacing between the conjugated antibodies was optimized, which minimizes steric hindrance and enhances antigen-antibody binding kinetics, leading to increased sensitivity, selectivity, and reproducibility. We used this DPA to profile protein phosphorylation in samples obtained from the brains of Alzheimer's disease (AD) mice model. The phosphorylation profiles provided the phosphorylations deregulated in the AD mouse brains and their associated signaling pathways.

## Results

### Conically-shaped dendron-coated surface

**Figure 1:**
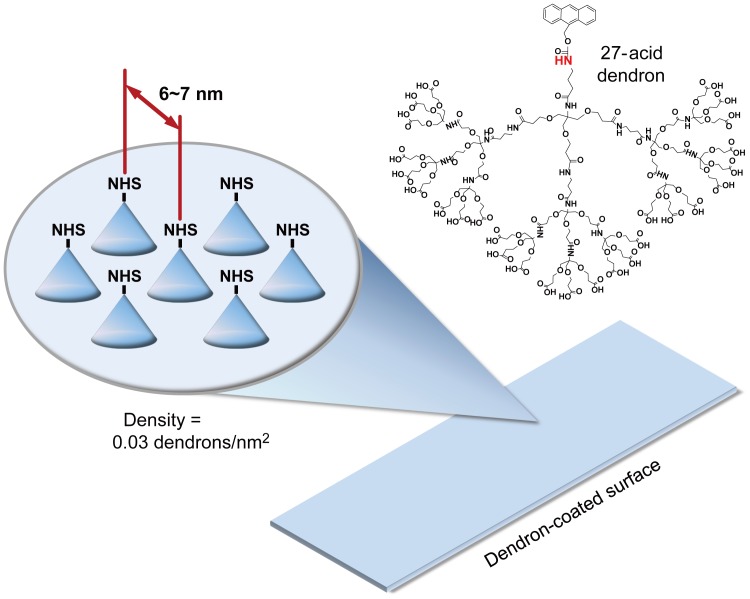
A dendron-coated surface. The 27-acid dendrons are coated on the surface. The chemical structure of the dendron is shown. After the immobilization and deprotection, the NH_2_ group at the apex is activated with DSC to generate NHS group. Through the deprotection, the NH group denoted in red becomes the primary amine. Phosphosite-specific antibodies are immobilized at the apex of dendron molecules through the amine reaction with the NHS group. A proper lateral spacing (6∼7 nm) of the immobilized antibodies is obtained. The dendron density on the surface was estimated to be 0.03 dendrons per nm^2^.

We previously reported that self-assembly of a conically shaped dendron on a glass slide provided controlled lateral spacing on the surface, and DNA microarrays prepared on the surface showed various advantages including high single nucleotide discrimination efficiency and short washing time. Also, enhanced sensitivity and selectivity [17] of the surface was demonstrated for detecting single nucleotide variations in the p53 gene [18]. In this study, we applied the same approach for a phosphokinase antibody array. We synthesized a third generation dendron in which 27 carboxylic groups are at the periphery and a chemically protected primary amine group is at the apex (**Fig. 1**) as previously described [17]. After the self-assembly of the dendron on the silanized surface, the surface was treated with trifluoroacetic acid to generate the primary amine group at the apex. Multiple ester bonds were formed between the carboxylic acids and the hydroxyl group on the silanized surface. Thus, each immobilized dendron occupied a certain area on the surface, and the occupation dictated the distance between the apexes or the primary amine groups. The surface density of the immobilized dendron was estimated to be 0.03 dendrons per nm^2^ based on the hexagonal close packing, and the corresponding lateral spacing was 6 to 7 nm. The lateral spacing on the dendron-coated glass slides provides sufficient space to accommodate the phosphosite-specific antibodies with the minimized steric hindrance.

### Immobilization of phosphosite-specific antibodies on the dendron-coated surface

**Figure 2:**
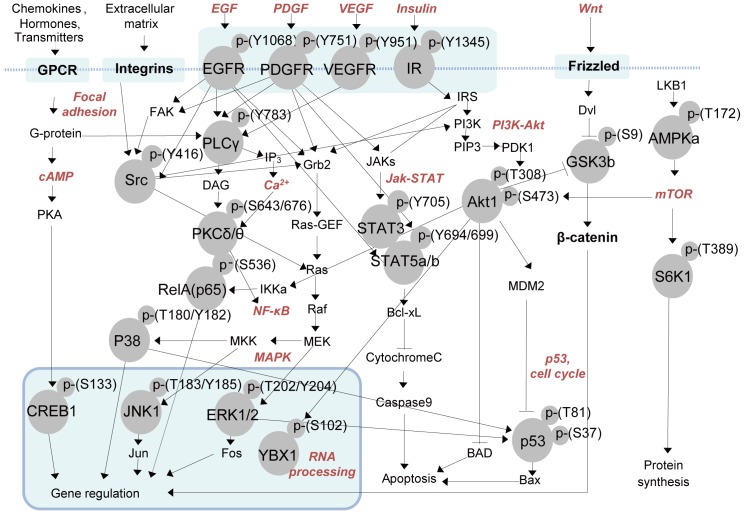
Signaling pathways represented by the proteins including 22 phosphosites. The components involved in the 15 pathways labeled in bold were obtained from KEGG [19] and BioCarta [20]. The 22 phosphosites were denoted as the circles while the signaling components in the 15 pathways were denoted as the labels. The number after the residue represents the residue position in the human protein containing the phosphosite (**Table S2**). The arrows represent activation while the inhibition symbols represent inactivation.

Key signaling pathways determining cellular physiology are often unknown. Monitoring a wide range of signaling pathways is important to identify key pathways for which detailed functional studies can be carried out. To systematically explore activities of intracellular signaling pathways, we first grouped 248 signaling pathways in KEGG [19] and BioCarta [20] databases into the representative pathways based on the pathway models in the KEGG database. We then selected 22 phosphosites (**Table S2**) that can characterize 15 representative pathways (**Fig. 2**): cAMP, calcium, JAK-STAT, PDGF, EGF, MAPK, PI3K-AKT, WNT, NF-κB, mTOR, P53, VEGF, insulin, RNA processing, and focal adhesion. We then selected the antibodies that are specific for the 22 phosphosites (**Table S2**). Rabbit antibodies generally known to have higher binding affinities than mouse antibodies, mostly monoclonal, were selected except the antibody specific for p-Y751 of PDGFR, which was not available. These 22 phosphosite-specific antibodies were printed on a 1″×3″ glass slide coated with the dendron using Genetix QArray-Mini microarray spotter (**Materials and Methods**). One DPA comprises two panels (left and right panels in **Fig. S1**), and each panel has two duplicates of the 22 antibodies, resulting in four replicate measurements per panel to ensure statistical power in data analysis.

### Evaluation of sensitivity and selectivity

**Figure 3:**
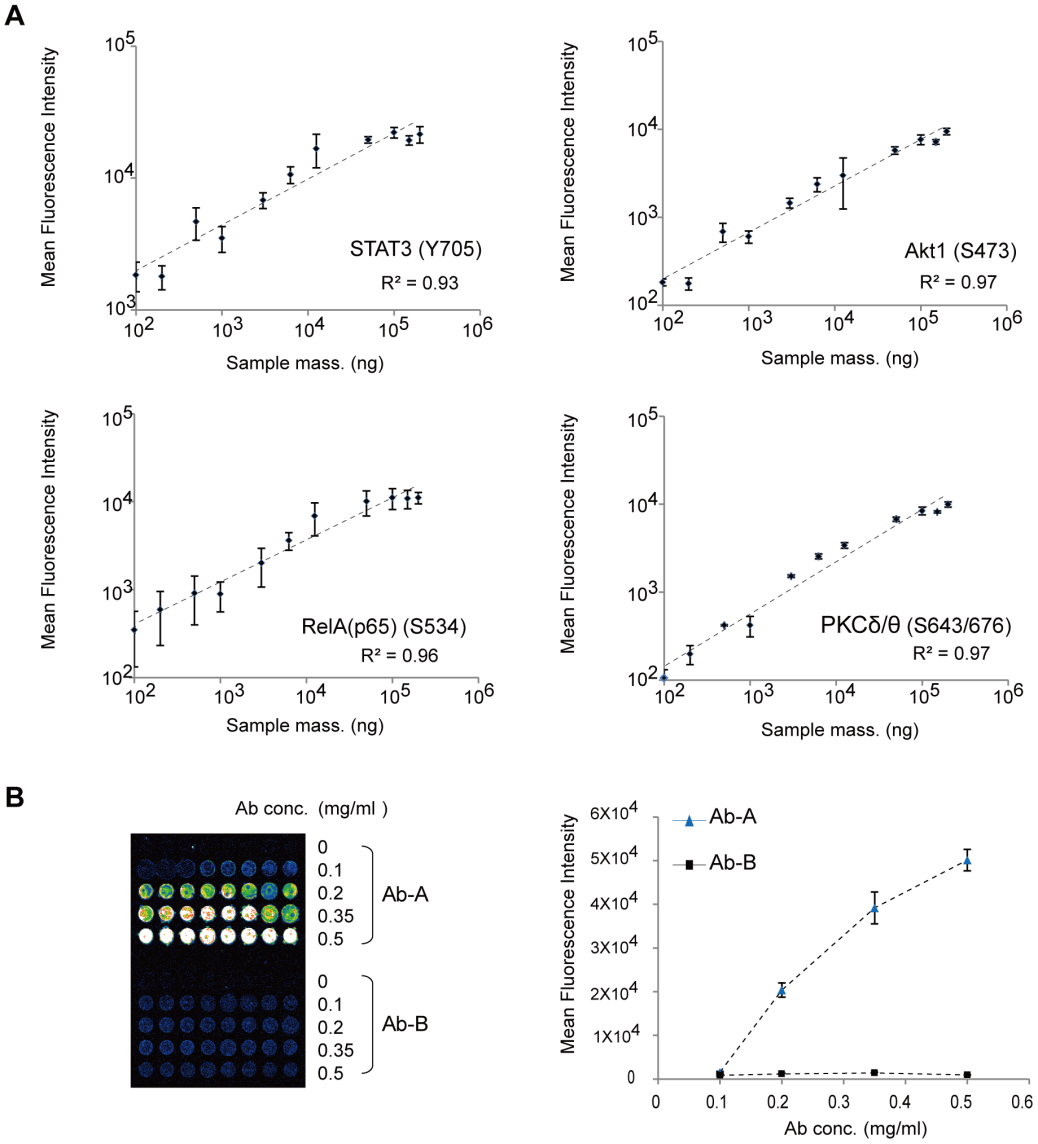
Evaluation of sensitivity and selectivity. **A**. Scatter plots between sample masses and measured intensities of four phosphosites in individual samples. The 11 sample masses hybridized onto the DPA in the ranges between 1.00×10^2^ and 2.00×10^5^ ng are shown (X-axis). Signal intensities of four technical replicates for the four phosphosites were plotted with mean ± standard deviation (Y-axis). For each selected antibody, a linear relationship was observed (R^2^>0.93). **B**. Scatter plots between amounts of the antibodies and measured intensities. For a fixed amount of the analyte (Apo-I), the amounts of the antibodies were varied (X-axis). The signal intensity (Y-axis) detected by Ab-A specific for Apo-I was apparent while the intensity detected by Ab-B was residual. The image corresponding to the signal intensities is also shown (left).

The quality of phosphokinase antibody arrays is determined by sensitivity of the signal intensities measured. Thus, we evaluated the sensitivity of the DPA by measuring signal intensities of four target phosphosites with varying the amounts of input samples. We labeled the proteins extracted from brain tissues of the littermate control mouse with Cy3, diluted the extracted proteins into 11 different sample masses from 1.00×10^2^ ng to 2.00×10^5^ ng (1.00×10^2^, 2.00×10^2^, 5.00×10^2^, 1.00×10^3^, 3.00×10^3^, 6.25×10^3^, 1.25×10^4^, 5.00×10^4^, 1.00×10^5^, 1.50×10^5^, and 2.00×10^5^ ng) with the final volume of 200 µl filled with PBST, and then hybridized each sample on the DPA. For all the tested sample masses, highly linear relationships (R^2^>0.93) between the intensities and the amounts of the proteins were observed in the sample mass range of 1.00×10^2^ ng to 2.00×10^5^ ng (**Fig. 3A**), indicating the detection sensitivity down to 1.00×10^2^ ng samples with the dynamic range of 10^3^. A key issue in protein antibody arrays is cross-reactivity that critically affects the selectivity [3]. To evaluate the cross-reactivity, we spotted different amounts of the antibodies (Ab-A and B) specific for Apo A-I and Apo B-100 in the concentration range of 0.1 and 0.5 mg/ml, hybridized the sample including only Apo A-I, and then measured the intensities of Apo A-I (**Fig. 3B**). Residual intensities were obtained for the wide range of Apo A-I concentrations, indicating that the high selectivity of the Ab-A antibody against Ab-B was acquired using the DPA. Moreover, the phosphosite-specificity of the 22 antibodies evaluated using western blotting analysis is summarized in **Table S3** and **Fig. S2**.

### Evaluation of reproducibility

Another key feature of phosphokinase antibody arrays is reproducibility of the signal intensities measured. Reproducibility of the signal intensity in protein antibody arrays is determined by the homogeneity of the surface, the spotting protocol, the mixing with the target sample, and others. Among these factors, we examined the variation in terms of the spot size and the signal intensity to assess the homogeneity of the surface and spotting determined by dendron coating and immobilization of antibodies. We applied DPA to fluorescent dye-labeled protein extracts from mouse brain tissues and obtained Cy3 fluorescence images. We first estimated the diameter distribution of the fluorescent spots using these images. The spot diameter is normally distributed with the mean of 196 µm (**Fig. S3A**). For each of the 22 phosphosite-specific antibodies, the coefficient variation (CV) of the spot diameter was also evaluated from the four replicates (**Fig. S1**). For 20 of 22 antibodies (90.9%), the CV was less than 5% with the maximum CV less than 10% (**Fig. S3B**), indicating well-controlled spot sizes. Moreover, to evaluate the regularity of the lateral spacing between the spots, we calculated the center-to-center distances between the 88 spots (**Fig. S3C**). The CV of the center-to-center distance normally distributed was found to be 1.2%. Furthermore, we also examined the variation of the intensities obtained from the four replicates for each of the 22 phosphosite-specific antibodies (**Fig. S3D**). For all the 22 antibodies, the CV was less than 10% (**Fig. S3D**). The normality of the spot diameter and the center-to-center distance, together with the small CVs of the spot diameter, the center-to-center distance, and the signal intensity, indicate that the dendron-coated surface was homogeneous, and the immobilization of the phosphosite-specific antibodies on the surface was well-controlled.

### Age-dependent changes in phosphorylation of signaling molecules in AD brain

**Figure 4:**
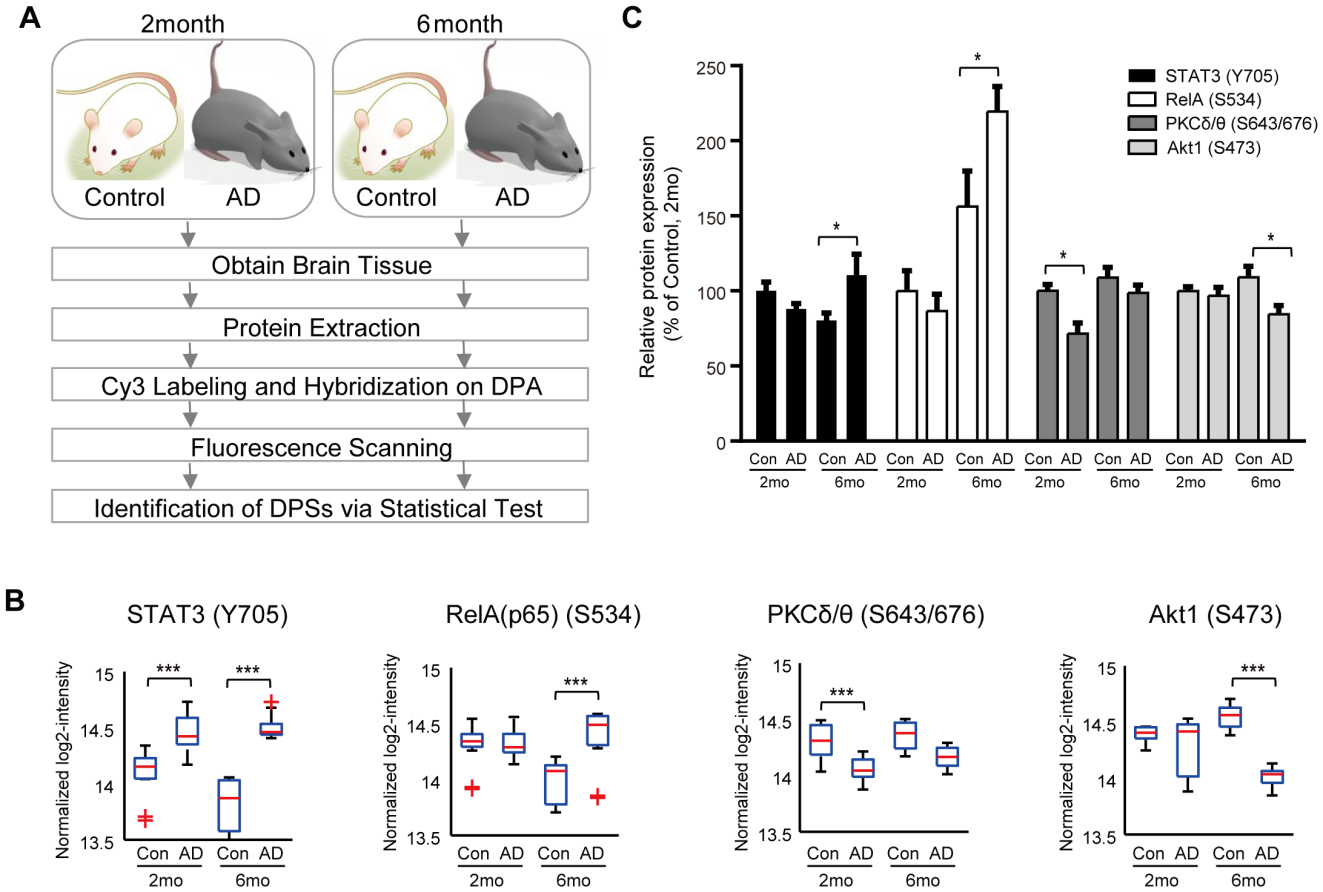
Application of DPA to AD brain tissues. **A**. Experimental scheme for phosphorylation profiling from AD brain tissues obtained from normal (Control) and AD mouse model (AD) at the ages of 2 and 6 months. **B**. Boxplots of the four DPSs whose alterations in AD, compared to control, were confirmed by Western blotting. ***P<0.001 from ANOVA followed by post-hoc tests with Bonferroni correction. **C**. Results of Western blotting of the four DPSs. Up-regulation of STAT3(Y705) and RelA(S534) at 6 months, and down-regulation of PKCδ/θ(S643/676) at 2 months and Akt1(S473) at 6 months were confirmed in independent samples (n = 3). Data are normalized to the β-actin abundance and presented as percentage changes from the control group. Data are shown as means ± SEM. *P<0.05 from ANOVA followed by Tukey's leat significant difference post-hoc tests.

To demonstrate the utility of the DPA, we applied it to investigate AD-dependent changes of signal transduction pathways using the AD mouse brain and also to monitor these alterations during the AD progression by comparing the results in different ages. We isolated the proteins from the brain tissues of normal (Control) and AD model mice (AD) at the ages of two and six months, respectively, labeled the isolated proteins with Cy3, and then hybridized the labeled proteins to the DPA (**Fig. 4A**). The Cy3 intensities were then measured. This procedure was repeated three times for independent control and AD samples. After normalizing the Cy3 intensities using the scale normalization method [21], we evaluated reproducibility of signal intensities within technical and biological replicates. For the four technical replicates in individual samples, the distributions of the CVs of the Cy3 intensities showed that the mean CVs of 22 phosphosites were 6.19% and 5.36% on average for the samples obtained at two and six months (**Table S4**), indicating high reproducibility of the signal intensities. We then identified three and seven differentially phosphorylated sites (DPSs) with P<0.01 between Control and AD at two months and six months, respectively, using the analysis of variance (ANOVA) followed by post-hoc tests with Bonferroni correction [22] (**Fig. 4B**; **Fig. S4**; **Table S5**; see **Materials and Methods** for detail). The DPSs represent the deregulation of signaling pathways in which they are possibly involved during the AD progression in AD mouse brain. Among the total of nine DPSs (3 and 7 DPSs in two and six months, respectively), the alterations of the six phosphorylation sites [STAT5a/b(Y694/Y699) [23], STAT3(Y705) [24], Akt1(S473) [25], JNK1(T183/Y185) [26], [27], GSK3b(S9) [28] and EGFR(Y1069) [29]] were previously shown to be associated with AD pathogenesis, which supports the validity of the DPA. In contrast, to our knowledge, the other three DPSs [Rela(S534), PKCδ/θ(S643/676), and Src(Y418)] have never been previously reported in association with AD pathogenesis, thus indicating their potential roles in AD pathogenesis. Finally, we confirmed the differential phosphorylation of the DPSs using Western blotting analysis in three independent samples under each condition. For this analysis, of the nine selected DPSs, we selected the two up-regulated phosphosites [STAT3(Y705) and Rela(S534)] and also the two down-regulated phosphosites [PKCδ/θ(S643/676) and Akt1(S473)] to include the two known and two unknown phosphorylations in association with AD pathogenesis (see above). All of the four showed the changes consistent to the ones detected by the DPA (**Fig. 4C**) except for up-regulation of STAT3(Y705) at 2 months probably due to the difference between solid-phase (DPA) and liquid-phase (Western blotting) assays.

### A network model for AD-mediated alteration in signaling pathways

**Figure 5:**
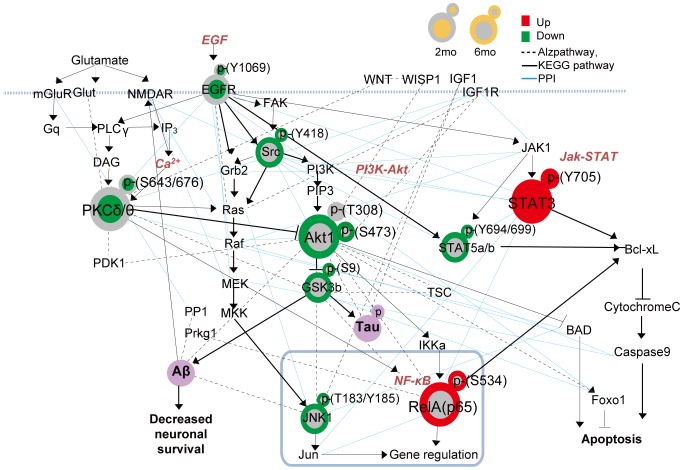
A network model describing perturbed signaling pathways in the AD brain. The nodes are arranged based on the signaling pathways in which the nine DPSs are involved (see text for the nine DPSs used for the network modeling). The node (center) and boundary colors represent the increase (red) and decrease (green) in phosphorylation measured by the DPA in AD samples at two and six months, respectively, compared to Control. The nodes corresponding to the four DPSs whose differential phosphorylation was confirmed by western blotting (STAT3, RelA, PKCδ/θ and Akt1) were denoted by large nodes. The arrows represent activation while the inhibition symbols represent inactivation. The dashed lines indicate interactions obtained from Alzpathway, and the solid lines indicate protein-protein interactions collected from BIND, HPRD, and BioGrid databases.

To understand the deregulation of signaling pathways associated with the DPSs, we reconstructed a signaling network model that delineates the relationships among the selected DPSs and their associated pathways (**Fig. 5**) during AD progression using the protein-protein interactions collected from AlzPathway [30], a comprehensive pathway map of AD, as well as BIND [31], HPRD [32], and BioGrid [33] databases (See **Materials and Methods** for detail). In this network modeling, we focused on the four DPSs confirmed by Western blotting, but also included the other five DPSs that were not tested by Western blotting. This network model showed 1) early down-regulation of PKCδ/θ(S643/676) and EGFR pathways and also 2) late changes in five pathways (up-regulation of STAT3 and NF-κB/Rela pathways and down-regulation of STAT5a/b, Akt-GSK3b, and JNK pathways). Among them, the Akt-GSK3b [34], [35] and JNK [26] pathways have been previously reported to modulate tau phosphorylation and thus neuronal cell death in AD brains. Also, PKC signaling pathways regulate production of the secretory form of amyloid precursor protein via activation of α-secretase activity, which reduces accumulation of pathogenic Aβ in the brain [36]. The Aβ was shown to activate NF-κB/Rela pathways in cultured neurons [37]. These data suggest potential implications of these altered pathways in AD pathogenesis. Furthermore, the network model showed that these pathways collectively contribute to AD pathogenesis with different dynamics during the AD progression. Conclusively, our findings demonstrate that the phosphosite profiling using the DPA provide a basis for designing detailed functional studies to investigate individual and collective roles of DPSs and associated pathways in AD pathogenesis.

## Discussion

Effective screening of key signaling pathways defining a cellular event is important when no clues on the pathways or only fragmented knowledge are available. Detailed mechanistic studies can then be followed for key pathways selected from the screening. MS-based phosphoproteomic analysis is still challenging for tissue samples obtained from human and animal models. Thus, protein antibody arrays have emerged as preferred tools for screening key signaling pathways in tissue samples. In this study, we developed the DPA that can be easily applied to tissue samples, as well as cell lines, for the effective screening of key signaling pathways. By systematically analyzing all signaling pathways in KEGG and BioCarta databases, we selected 22 phosphosites that can be used to monitor the activities of 15 representative pathways. Although commercial phosphokinase antibody arrays are available to provide phosphorylation profiles to monitor the activities of similar or more signaling pathways, they require high expenses for screening key signaling pathways. Thus, compared to these commercial arrays, the DPA can provide rapid, cost-effective phosphorylation profiling of the 15 pathways for screening key signaling pathways.

The DPA, compared to the commercial arrays, has special features including surface coating of conically-shaped dendrons on a glass slide and covalent conjugation of phosphosite-specific antibodies onto the apexes of the dendrons. We previously demonstrated that the dendron-coated surface provides the apexes of the dendrons with nano-scale spacing that minimized the steric hindrance of the immobilized biomolecules. A NH_2_- (or NHS-) functional group at the apex allowed the facile specific binding of the antibodies through the strong covalent bond. Therefore, low concentration of the antibodies was sufficient enough for the spotting. It is expected that most of the antibodies are conjugated through the Fc region, because the active amine is abundant in the region. The epitopes of the antibodies should be upwards to interact with the corresponding antigens effectively. The orientation control as well as the controlled lateral spacing between the immobilized antibodies promise the enhanced binding affinity and kinetics. Thus, these special features of the DPA provide high sensitivity and selectivity, as well as reproducibility, as previously demonstrated in other applications of the dendron-coated surface [17], [18] and also in this study.

AD is one of the devastating neuropathological diseases, and diverse perturbations in signaling pathways have been reported, including altered phosphorylations of signaling molecules. Through the application of the DPA to brain tissues from AD model mice, we reconstructed a dynamic signaling network model that shows early and late perturbation of seven pathways during the course of AD progression. The network model showed the early and late alterations of the pathways associated with the four DPSs confirmed by Western blotting [early down-regulation of PKCδ/θ pathway, late up-regulation of STAT3(Y705) and RelA(S534), and late down-regulation of Akt1-GSK3b pathway]. The combinatorial alteration state of the four DPSs and their associated pathways can be proposed as a metric to monitor the disease activity during AD progression. The network model further suggested individual and collective roles of these pathways in AD pathogenesis. The roles of individual pathways and their interplays can be subjects for detailed mechanistic studies. In summary, the DPA serves as a useful tool for an effective screening of key signaling pathways and for developing a signaling network model describing the relationships among the key pathways, thereby providing hypotheses for the detailed functional studies.

## Materials and Methods

### Antibodies

All antibodies used for the DPA were commercially obtained (Cell Signaling Technology, Inc., Danvers, MA). List of 22 antibodies, catalog numbers (CST#), their reactivity in human and/or mouse, phosphosites, and their associated pathways are shown in **Table S2**. Also, the phosphosite-specificity of the 22 antibodies are summarized in **Table S3** and **Fig. S2**. Purified human plasma Apo A-I was obtained from Sigma. The Apo-A-I antibody and Apo B-100 antibody used for evaluation of sensitivity and selectivity were obtained as previously described [38]–[41].

### Preparation of glass slides

Dendron-modified glass slides (NSB27 NHS Slide) were prepared as previously described [42] by using a standard (1″×3″) glass slide (Schott NEXTERION Glass B, ultrasonically cleaned, #1055132). The slides were purchased (NSB POSTECH, Korea) and used for the phosphokinase antibody array. Briefly, (9-anthrylmethyl-3-({[tris-({[(1-{tris[(2-{[(tris{[2-carboxyethoxy]methyl}methyl)amino]carbonyl}ethoxy) methyl]-methyl}amino)carbonyl]-2-ethoxy}methyl)methyl]amino}carbonyl) propylcarbamate, called 27-acid dendron, was introduced onto silylated glass slide through multiple covalent bond formation. After removal of the protecting group at the apex of dendron, *N,N'*-disuccinimidyl carbonate (DSC) was conjugated to the generated NHS group as a crosslinker.

### Printing phosphosite-specific antibodies onto slides

Antibodies in a printing buffer (0.01% BSA, 0.01% Tween 20, 0.5% glycerol in 1× PBS buffer (pH 8.5)) were spotted on the NHS-activated NSB27 slides by Genetix QArray-Mini microarray spotter. After the printing, the printed slides were incubated in a 70∼85% humidified chamber for 5∼12 hrs and then washed in 1× PBST (Sigma P3563-10PAK) and subsequently in 1× PBS (Sigma P4417-100TAB). Any chance of the printed slides getting dried in the process of washing was avoided. For storage, the slides, together with a moisturized cotton pad, were put in a slide container and the container was sealed (with no evacuation, no desiccant inside). In our experiences, the microarray slides stored in this way at −20°C was active for up to one year, whereas the activity was maintained for 3–4 weeks when stored at 4°C.

### Protein extraction

Transgenic mice with 5XFAD used in this study were purchased from The Jackson Laboratory (Bar Harbor, ME, USA). These mice overexpressing Aβ peptide express both mutant human amyloid precursor protein (695) (with the Swedish mutation (K670N, M671L), Florida mutation (I716V), and London mutation (V717I)) and human presenilin 1, harboring two familial Alzheimer's disease (FAD) mutations (M146L and L286V) [43]. Male animals were killed at 2 or 6 months of age (n = 3). The cortical regions were used, and the tissues were solubilized with RIPA buffer which contains protease inhibitor, phophatase inhibitor, and phenylmethane-sulfonylfluoride (PMSF). For serial dilution of the protein extracts, we quantified the proteins extracted from the mouse brain tissues using Pierce BCA protein assay kit (Thermo Scientific Cat. 23225, Rockford, USA). Animals were maintained and procedures were performed in Seoul National University's mouse facility. All experiments were approved by the Institute of Laboratory Animal Resources of Seoul National University, Seoul, Korea. All procedures were made to minimize the number of animals used and animal suffering, and approved by the Ethics Review Committee for Animal Experimentation in Seoul National University (approval number, SNU060519-5, IACUC).

### Protein labeling and hybridization

Cy3 dyes were labeled to extracted proteins using Amersham CyDye mono-Reactive NHS Ester (GE Healthcare Cat. PA13101). The labeled proteins were purified by centrifugation through Amicon Ultra-0.5 mL Centrifugal Filters, 10 kDa (Millipore Cat. UFC501096). Before the hybridization, to reduce nonspecific binding, we added 200 µl of a blocking buffer with 3% Block Ace (SCETI K.K., Japan, Cat. UK-B80) in PBST to each chamber of Agilent 2 chamber gasket slides (Cat. G2534-60002) and then incubated the slides for 4 hrs with rotation. The slides were washed with PBST and incubated for 2 hrs with 25 µg of Cy3-labeled proteins in a final volume of 200 µl filled with PBST. After a series of thorough washing with PBST, PBS, and ddH_2_O, the slides were dried and the Cy3 fluorescence intensities were obtained by a confocal laser scanner (GenePix 4000B, Molecular Devices). We deposited the DPA data generated in this study at the NCBI Gene Expression Omnibus under accession GSE55452.

### Identification of DPSs

We converted signal intensities from the array experiment into log_2_-intensities and normalized it using the scale normalization method as previously reported [21]. We then applied ANOVA followed by post-hoc tests with Bonferroni correction [22] to identify DPSs between control and AD samples at 2 and 6 months, respectively. We identified the DPSs as the ones with P<0.01 from the post-hoc tests with Bonferroni correction. To further reduce false positives, we selected the DPSs showing significant fold-changes (P<0.05 from median fold-change test previously reported [22]; **Fig. S4**) and the reactivity in the species used (mouse in this study; **Table S2**).

### Western blotting

Western blotting was performed as described previously [44]. Briefly, brain lysates were resuspended with RIPA buffer (50 mM Tris-HCl, 150 mM NaCl, 1% Nonidet P-40 (NP-40), 0.1% SDS, 0.5% Deoxicholic acid sodium salt, pH 7.4)-containing protease inhibitor cocktail (Sigma-Aldrich). After centrifugation at 13,000 rpm for 15 min, the supernatant was collected, and protein concentration was determined by a BCA assay kit (Pierce, Rockford, IL, USA). For western blotting analysis, equal amounts of protein (40 ug per lane) were loaded on each Tris-glycine polyacrylamide gel, and the immunoreactive bands were photographed and quantified on an LAS-3000 with MultiGauge (FujiFilm Inc.).

### Reconstruction of a signaling network

We mapped the nine DPSs onto protein-protein interaction (PPI) maps collected from AlzPathway [30], a comprehensive map of signaling pathways collected from AD-related literatures. We extracted a subnetwork containing the nine DPSs and their first neighbors and then added PPIs among the DPSs and their first neighbors using the PPIs obtained from BIND [31], HPRD [32], and BIOGRID [33]. We arranged the nodes in the subnetwork based on the KEGG pathways in which they are involved and grouped them by their associated pathways. Nodes not assigned into the functional group were removed from the subnetwork.

## Supporting Information

Figure S1 **Two panels in a DPA. Each panel contains the spots for two technical replicates of 22 phosphosites.** See **Table S2** for the labels of phosphosite-specific antibodies.
(TIF)

Figure S2 **Phosphosite-specificity of the 22 antibodies on DPS. A–V.** Western blot images to evaluate the phosphosite-specificity of the 22 antibodies in human samples collected from the Cell Signaling Technology. Western blotting data show that each antibody used detects specifically the changes in its target phosphorylation levels when the stimulation previously reported to alter the target phosphorylation is applied, compared to the antibody that recognizes the whole protein. For example, **Fig. S2A** shows that the Src(Y416)-specific antibody detect specifically the changes in the phosphorylation levels of Src at Y416 in serum starved COLO 201 cells when the cells are stimulated with 20% FBS for 5 minutes (upper). By contrast, the antibody Ab #2110 that recognizes the whole Src protein does not detects the phosphorylation changes at Y416 (lower). Note that the positions of the phosphosites in **Fig. S2** differ from the ones in the text because the phosphosite-specificity data shown here are obtained from human samples, while the phosphosites in this study are detected from mouse samples (**Table S2**). The experimental designs to evaluate the phosphosite-specificity by Western blotting for the 22 antibodies were summarized in **Table S3**.
(PDF)

Figure S3 **Evaluation of reproducibility.** **A**. Distribution of spot diameters with CV = 4.1%. **B**. Distribution of CVs of spot diameters for 22 phosphosite-specific antibodies. **C**. Distribution of center-to-center distances among the spots with CV = 1.2%. **D**. Distribution of CVs of signal intensities for the replicates of 22 phosphosite-specific antibodies (4 replicates per antibody).
(TIF)

Figure S4 **Identification of the nine DPSs.** **A**. Overall scheme for statistical testing to identify the nine DPSs and reconstruction of a signaling network model for the DPSs. **B**. Boxplots of the five identified DPSs not shown in **Fig. 4B**. **, P<0.01 and ***, P<0.001 from ANOVA followed by post-hoc tests with Bonferroni correction.
(TIF)

Table S1 **Characteristics of commercial phosphokinase antibody arrays.** The information for five commercial phosphokinase antibody arrays is summarized. ^*^The number of phosphosite-specific antibodies in each array is as of April 2013.
(PDF)

Table S2 **Properties of antibodies spotted on the DPA.** The information for the 22 phosphosite-specific antibodies is summarized. All the 22 antibodies (catalog number: CST#) were purchased from Cell Signaling Technology. The residues of S, T, and Y represent serine, threonine, and tyrosine, respectively. The number after the residue represents the residue position in the corresponding protein. Phosphosite(H,M) indicates the position of the phosphorylation residue in human (H) and mouse (M) based on the PhosphositePlus database [45]. The pathways indicate the ones in which the corresponding protein is involved. ‘Host/Clonality’ represents the hosts from which mono- or poly-antibodies were generated. The reactivity of H or M indicates whether the antibodies are active in human (H) and mouse (M).
(PDF)

Table S3 **Phosphosite-specificity information of the 22 antibodies on DPA.** Experimental design for Western blotting to evaluate the phosphosite-specificity of each antibody were summarized from the documents provided by Cell signaling Technology.
(PDF)

Table S4 **Percentage CVs of signal intensities for the 22 phosphosites in individual samples and standard deviations (SD) of the 22 phosphosites.** In each sample, the intensities of four technical replicates for each phosphosite were used to compute the percentage CV. For each phosphosite, the pooled standard deviation was computed using the data of the three biological replicates each of which has the four technical replicates.
(PDF)

Table S5 **Statistical significance of the nine selected DPSs.** For each of the nine DPSs, the direction of phosphorylation changes (U: up-regulated, D: down-regulated in AD compared to control), p-value from the post-hoc test with Bonferroni correction after ANOVA test, p-value from the median fold-change test, and log_2_-fold-change at two and six months are shown.
(PDF)

## References

[1] Ficarro SB, McCleland ML, Stukenberg PT, Burke DJ, Ross MM, et al (2002) Phosphoproteome analysis by mass spectrometry and its application to Saccharomyces cerevisiae. Nat Biotechnol 20: 301–305. 11875433 10.1038/nbt0302-301

[2] Cutillas PR, Geering B, Waterfield MD, Vanhaesebroeck B (2005) Quantification of gel-separated proteins and their phosphorylation sites by LC-MS using unlabeled internal standards: analysis of phosphoprotein dynamics in a B cell lymphoma cell line. Mol Cell Proteomics 4: 1038–1051. 15879432 10.1074/mcp.M500078-MCP200

[3] Zhang H, Pelech S (2012) Using protein microarrays to study phosphorylation-mediated signal transduction. Semin Cell Dev Biol 23: 872–882. 22706299 10.1016/j.semcdb.2012.05.009

[4] Wolf-Yadlin A, Hautaniemi S, Lauffenburger DA, White FM (2007) Multiple reaction monitoring for robust quantitative proteomic analysis of cellular signaling networks. Proc Natl Acad Sci U S A 104: 5860–5865. 17389395 10.1073/pnas.0608638104 PMC1851582

[5] Angenendt P, Glokler J, Sobek J, Lehrach H, Cahill DJ (2003) Next generation of protein microarray support materials: evaluation for protein and antibody microarray applications. J Chromatogr A 1009: 97–104. 13677649 10.1016/s0021-9673(03)00769-6

[6] Chandra H, Reddy PJ, Srivastava S (2011) Protein microarrays and novel detection platforms. Expert Rev Proteomics 8: 61–79. 21329428 10.1586/epr.10.99

[7] Chan SM, Ermann J, Su L, Fathman CG, Utz PJ (2004) Protein microarrays for multiplex analysis of signal transduction pathways. Nat Med 10: 1390–1396. 15558056 10.1038/nm1139

[8] Paweletz CP, Charboneau L, Bichsel VE, Simone NL, Chen T, et al (2001) Reverse phase protein microarrays which capture disease progression show activation of pro-survival pathways at the cancer invasion front. Oncogene 20: 1981–1989. 11360182 10.1038/sj.onc.1204265

[9] Nishizuka S, Charboneau L, Young L, Major S, Reinhold WC, et al (2003) Proteomic profiling of the NCI-60 cancer cell lines using new high-density reverse-phase lysate microarrays. Proc Natl Acad Sci U S A 100: 14229–14234. 14623978 10.1073/pnas.2331323100 PMC283574

[10] Xiao K, Sun J, Kim J, Rajagopal S, Zhai B, et al (2010) Global phosphorylation analysis of beta-arrestin-mediated signaling downstream of a seven transmembrane receptor (7TMR). Proc Natl Acad Sci U S A 107: 15299–15304. 20686112 10.1073/pnas.1008461107 PMC2930550

[11] Allaeys I, Marceau F, Poubelle PE (2013) NLRP3 promotes autophagy of urate crystals phagocytized by human osteoblasts. Arthritis Res Ther 15: R176. 24456929 10.1186/ar4365 PMC4061723

[12] El-Haibi CP, Singh R, Gupta P, Sharma PK, Greenleaf KN, et al (2012) Antibody Microarray Analysis of Signaling Networks Regulated by Cxcl13 and Cxcr5 in Prostate Cancer. J Proteomics Bioinform 5: 177–184. 24009409 10.4172/jpb.1000232 PMC3760521

[13] Mayya V, Han DK (2009) Phosphoproteomics by mass spectrometry: insights, implications, applications and limitations. Expert Rev Proteomics 6: 605–618. 19929607 10.1586/epr.09.84 PMC2931417

[14] Benters R, Niemeyer CM, Wohrle D (2001) Dendrimer-activated solid supports for nucleic acid and protein microarrays. Chembiochem 2: 686–694. 11828505 10.1002/1439-7633(20010903)2:9<686::AID-CBIC686>3.0.CO;2-S

[15] Cretich M, Pirri G, Damin F, Solinas I, Chiari M (2004) A new polymeric coating for protein microarrays. Anal Biochem 332: 67–74. 15301950 10.1016/j.ab.2004.05.041

[16] Dai J, Baker GL, Bruening ML (2006) Use of porous membranes modified with polyelectrolyte multilayers as substrates for protein arrays with low nonspecific adsorption. Anal Chem 78: 135–140. 16383320 10.1021/ac0513966

[17] Hong BJ, Sunkara V, Park JW (2005) DNA microarrays on nanoscale-controlled surface. Nucleic Acids Res 33: e106. 16002785 10.1093/nar/gni109 PMC1174902

[18] Oh SJ, Ju J, Kim BC, Ko E, Hong BJ, et al (2005) DNA microarrays on a dendron-modified surface improve significantly the detection of single nucleotide variations in the p53 gene. Nucleic Acids Res 33: e90. 15939931 10.1093/nar/gni087 PMC1143581

[19] Kanehisa M, Goto S, Sato Y, Furumichi M, Tanabe M (2012) KEGG for integration and interpretation of large-scale molecular data sets. Nucleic Acids Res 40: D109–114. 22080510 10.1093/nar/gkr988 PMC3245020

[20] BioCarta website. Available: http://www.biocarta.com. Accessed 2014 Apr 10.

[21] Yang YH, Dudoit S, Luu P, Lin DM, Peng V, et al (2002) Normalization for cDNA microarray data: a robust composite method addressing single and multiple slide systematic variation. Nucleic Acids Res 30: e15. 11842121 10.1093/nar/30.4.e15 PMC100354

[22] Chae S, Ahn BY, Byun K, Cho YM, Yu MH, et al (2013) A systems approach for decoding mitochondrial retrograde signaling pathways. Sci Signal 6: rs4. 23443683 10.1126/scisignal.2003266

[23] Marwarha G, Prasanthi JR, Schommer J, Dasari B, Ghribi O (2011) Molecular interplay between leptin, insulin-like growth factor-1, and beta-amyloid in organotypic slices from rabbit hippocampus. Mol Neurodegener 6: 41. 21651786 10.1186/1750-1326-6-41 PMC3121598

[24] Wan J, Fu AK, Ip FC, Ng HK, Hugon J, et al (2010) Tyk2/STAT3 signaling mediates beta-amyloid-induced neuronal cell death: implications in Alzheimer's disease. J Neurosci 30: 6873–6881. 20484629 10.1523/JNEUROSCI.0519-10.2010 PMC6632652

[25] Ho L, Qin W, Pompl PN, Xiang Z, Wang J, et al (2004) Diet-induced insulin resistance promotes amyloidosis in a transgenic mouse model of Alzheimer's disease. FASEB J 18: 902–904. 15033922 10.1096/fj.03-0978fje

[26] Ploia C, Antoniou X, Sclip A, Grande V, Cardinetti D, et al (2011) JNK plays a key role in tau hyperphosphorylation in Alzheimer's disease models. J Alzheimers Dis 26: 315–329. 21628793 10.3233/JAD-2011-110320

[27] Sze CI, Su M, Pugazhenthi S, Jambal P, Hsu LJ, et al (2004) Down-regulation of WW domain-containing oxidoreductase induces Tau phosphorylation in vitro. A potential role in Alzheimer's disease. J Biol Chem 279: 30498–30506. 15126504 10.1074/jbc.M401399200

[28] Dewachter I, Ris L, Jaworski T, Seymour CM, Kremer A, et al (2009) GSK3beta, a centre-staged kinase in neuropsychiatric disorders, modulates long term memory by inhibitory phosphorylation at serine-9. Neurobiol Dis 35: 193–200. 19379814 10.1016/j.nbd.2009.04.003

[29] Wang L, Chiang HC, Wu W, Liang B, Xie Z, et al (2012) Epidermal growth factor receptor is a preferred target for treating amyloid-beta-induced memory loss. Proc Natl Acad Sci U S A 109: 16743–16748. 23019586 10.1073/pnas.1208011109 PMC3478595

[30] Mizuno S, Iijima R, Ogishima S, Kikuchi M, Matsuoka Y, et al (2012) AlzPathway: a comprehensive map of signaling pathways of Alzheimer's disease. BMC Syst Biol 6: 52. 22647208 10.1186/1752-0509-6-52 PMC3411424

[31] Bader GD, Betel D, Hogue CW (2003) BIND: the Biomolecular Interaction Network Database. Nucleic Acids Res 31: 248–250. 12519993 10.1093/nar/gkg056 PMC165503

[32] Peri S, Navarro JD, Amanchy R, Kristiansen TZ, Jonnalagadda CK, et al (2003) Development of human protein reference database as an initial platform for approaching systems biology in humans. Genome Res 13: 2363–2371. 14525934 10.1101/gr.1680803 PMC403728

[33] Chatr-Aryamontri A, Breitkreutz BJ, Heinicke S, Boucher L, Winter A, et al (2013) The BioGRID interaction database: 2013 update. Nucleic Acids Res 41: D816–823. 23203989 10.1093/nar/gks1158 PMC3531226

[34] Fang X, Yu SX, Lu Y, Bast RC Jr, Woodgett JR, et al (2000) Phosphorylation and inactivation of glycogen synthase kinase 3 by protein kinase A. Proc Natl Acad Sci U S A 97: 11960–11965. 11035810 10.1073/pnas.220413597 PMC17277

[35] Hernandez F, Lucas JJ, Avila J (2013) GSK3 and tau: two convergence points in Alzheimer's disease. J Alzheimers Dis 33 Suppl 1S141–144. 22710914 10.3233/JAD-2012-129025

[36] Kim T, Hinton DJ, Choi DS (2011) Protein kinase C-regulated abeta production and clearance. Int J Alzheimers Dis 2011: 857368. 21274428 10.4061/2011/857368 PMC3026967

[37] Mattson MP, Camandola S (2001) NF-kappaB in neuronal plasticity and neurodegenerative disorders. J Clin Invest 107: 247–254. 11160145 10.1172/JCI11916 PMC199201

[38] Kwak J-W CT, Han MH (1998) New monoclonal antibodies against human plasma apolipoprotein A-I and recombinant hybridoma cells for the production of them. Patent

[39] Kwak J-W HM, Choi BK (1999) cDNAs encoding murine antibody against human plasma apolipoprotein B-100.

[40] Kwak JW, Lee DI, Choi BK, Cho WK, Lee SH, et al (1996) Cloning and characterization of cDNAs coding for heavy and light chains of a monoclonal antibody (MabA34) specific for human plasma apolipoprotein A-I. Gene 173: 257–259. 8964510 10.1016/0378-1119(96)00077-7

[41] Kwak JW, Choi BK, Lee DI, Kang YK, Seo YG, et al (1996) Cloning and characterization of cDNAs coding for heavy and light chains of a monoclonal antibody (MabB23) specific for human plasma apolipoprotein B-100. Gene 169: 237–239. 8647454 10.1016/0378-1119(95)00807-1

[42] Cahill DJ (2001) Protein and antibody arrays and their medical applications. J Immunol Methods 250: 81–91. 11251223 10.1016/s0022-1759(01)00325-8

[43] Oakley H, Cole SL, Logan S, Maus E, Shao P, et al (2006) Intraneuronal beta-amyloid aggregates, neurodegeneration, and neuron loss in transgenic mice with five familial Alzheimer's disease mutations: potential factors in amyloid plaque formation. J Neurosci 26: 10129–10140. 17021169 10.1523/JNEUROSCI.1202-06.2006 PMC6674618

[44] Son SM, Jung ES, Shin HJ, Byun J, Mook-Jung I (2012) Abeta-induced formation of autophagosomes is mediated by RAGE-CaMKKbeta-AMPK signaling. Neurobiol Aging 33: : 1006 e1011–1023. 10.1016/j.neurobiolaging.2011.09.039 22048125

[45] Hornbeck PV, Kornhauser JM, Tkachev S, Zhang B, Skrzypek E, et al (2012) PhosphoSitePlus: a comprehensive resource for investigating the structure and function of experimentally determined post-translational modifications in man and mouse. Nucleic Acids Res 40: D261–270. 22135298 10.1093/nar/gkr1122 PMC3245126

