# Supplementary figures and images for: Phosphokinase Antibody Arrays on Dendron-Coated Surface
Source: PLoS One. 2014 May 6;9(5):e96456. doi: 10.1371/journal.pone.0096456 (PMC4011796; doi:10.1371/journal.pone.0096456)

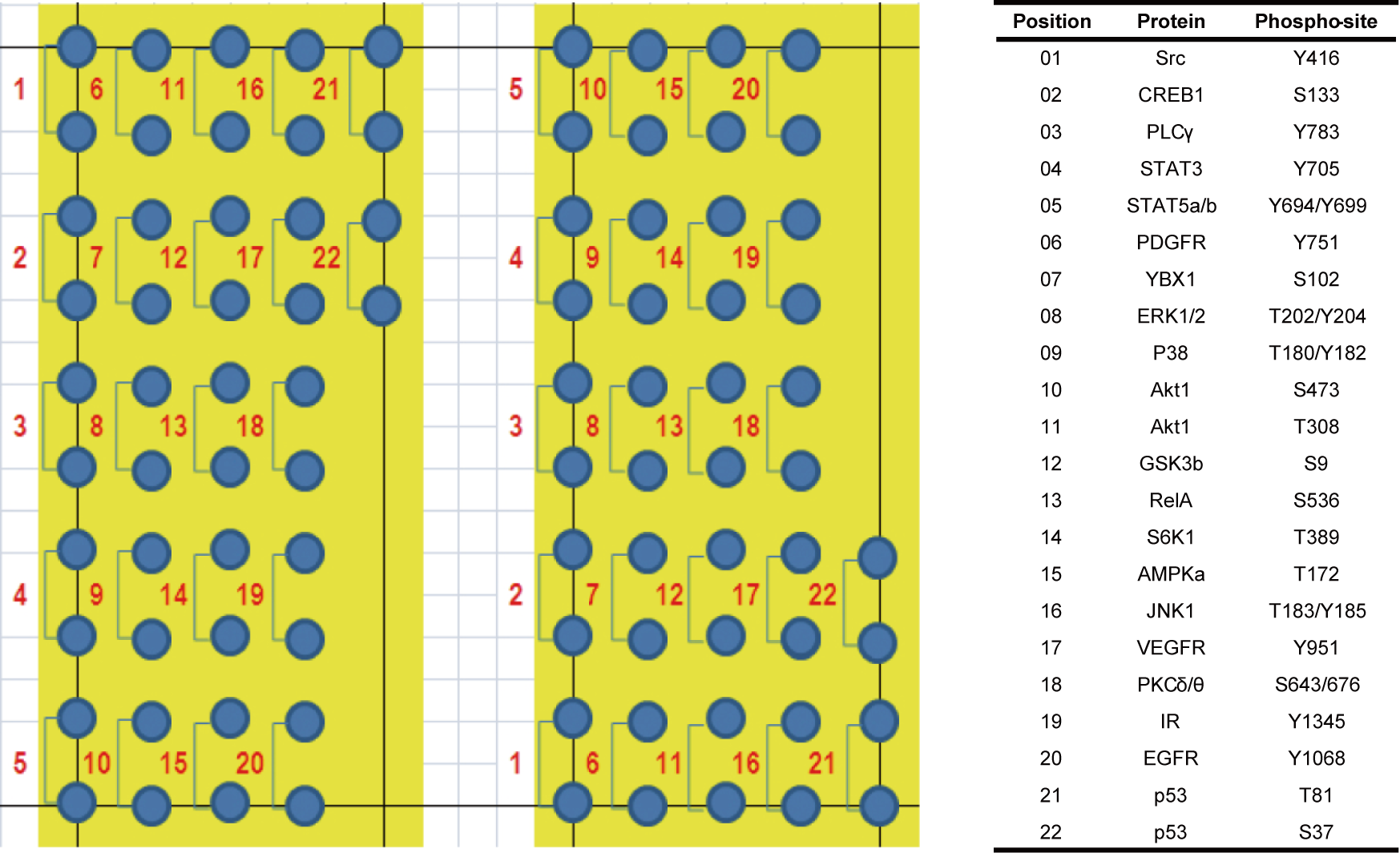

Supplement: Figure S1 — Two panels in a DPA. Each panel contains the spots for two technical replicates of 22 phosphosites. See Table S2 for the labels of phosphosite-specific antibodies. (TIF) [file pone.0096456.s001.tif]

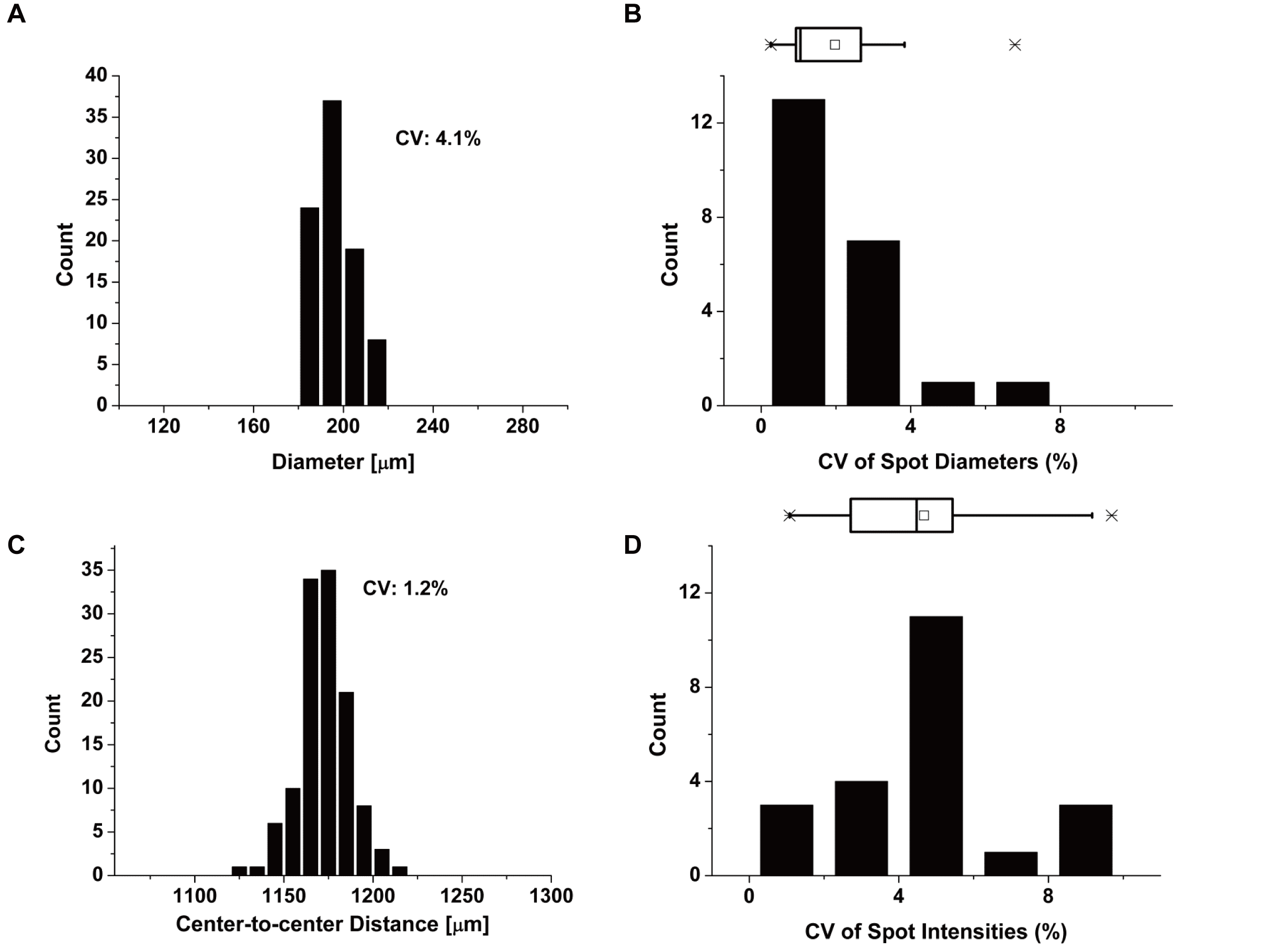

Supplement: Figure S3 — Evaluation of reproducibility. A. Distribution of spot diameters with CV = 4.1%. B. Distribution of CVs of spot diameters for 22 phosphosite-specific antibodies. C. Distribution of center-to-center distances among the spots with CV = 1.2%. D. Distribution of CVs of signal intensities for the replicates of 22 phosphosite-specific antibodies (4 replicates per antibody). (TIF) [file pone.0096456.s003.tif]

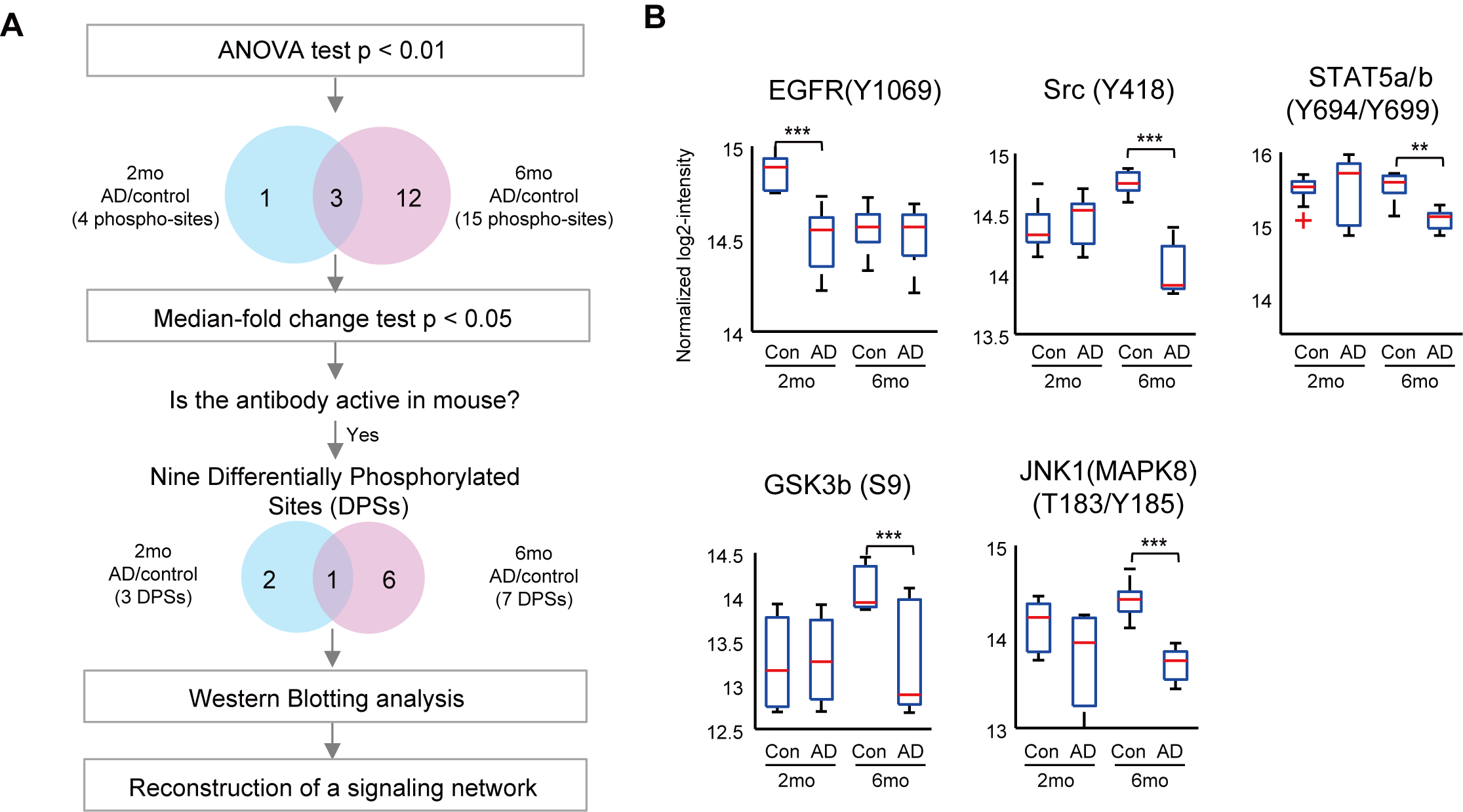

Supplement: Figure S4 — Identification of the nine DPSs. A. Overall scheme for statistical testing to identify the nine DPSs and reconstruction of a signaling network model for the DPSs. B. Boxplots of the five identified DPSs not shown in Fig. 4B . **, P<0.01 and ***, P<0.001 from ANOVA followed by post-hoc tests with Bonferroni correction. (TIF) [file pone.0096456.s004.tif]
