# Supplementary material for: Phosphokinase Antibody Arrays on Dendron-Coated Surface
Source: PLoS One. 2014 May 6;9(5):e96456. doi: 10.1371/journal.pone.0096456 (PMC4011796; doi:10.1371/journal.pone.0096456)

A. Src (Y416), CST#2113

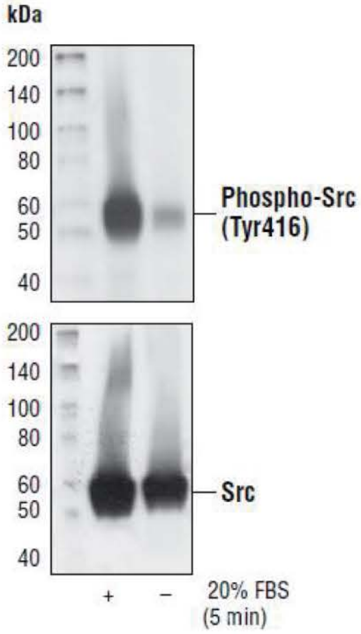

B. CREB1 (S133), CST#9198

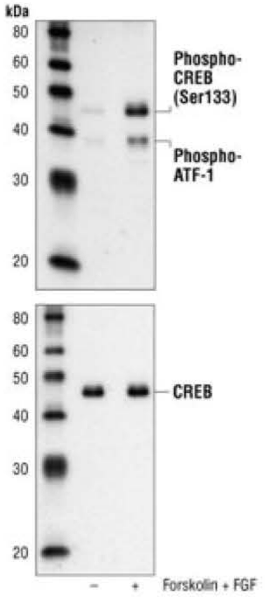

C. PLCγ (Y783), CST#2821

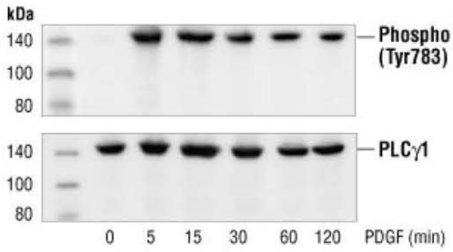

D. STAT3 (Y705), CST#9145

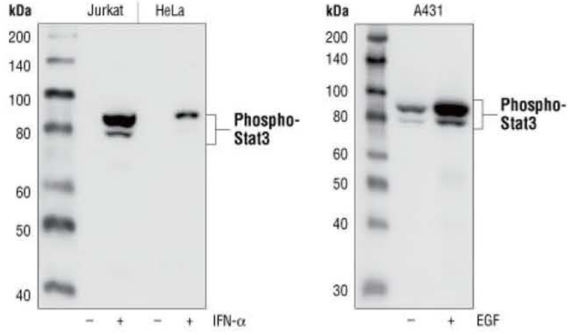

E. STAT5a/b (Y694/699), CST#4322

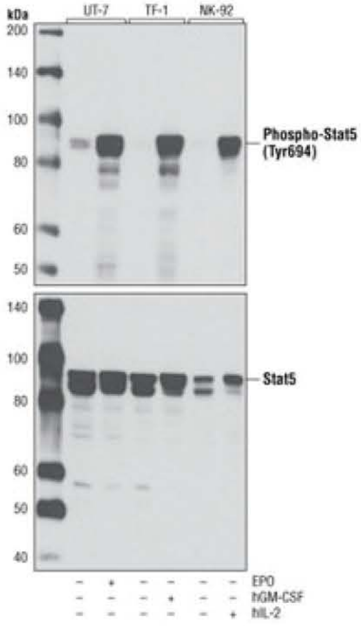

F. PDGFR (Y751), CST#3166

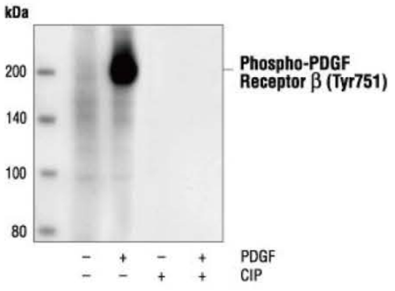

G. YBX1 (S102), CST#2900

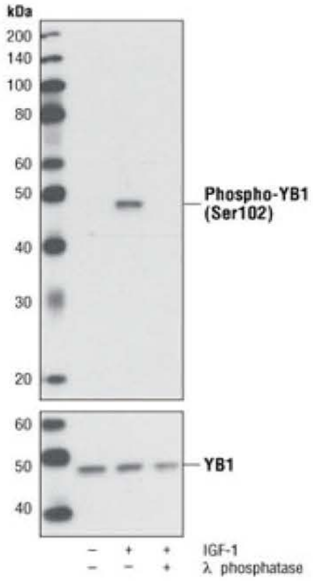

H. ERK1/2 (T202/Y204), CST#4377

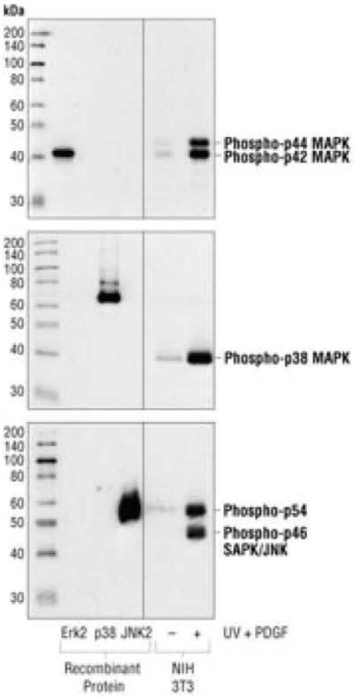

I. P38 (T180/Y182), CST#4511

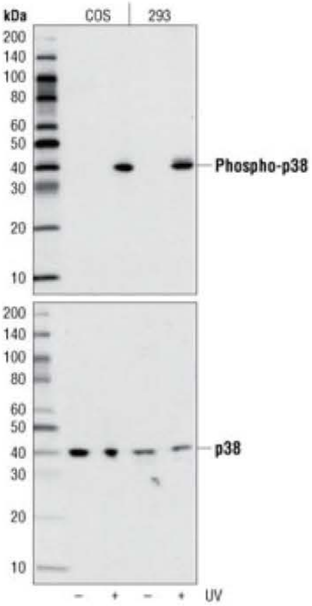

J. Akt1 (S473), CST#4058

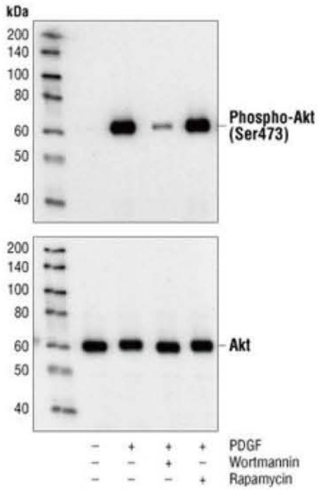

K. Akt1(T308), CST#2965

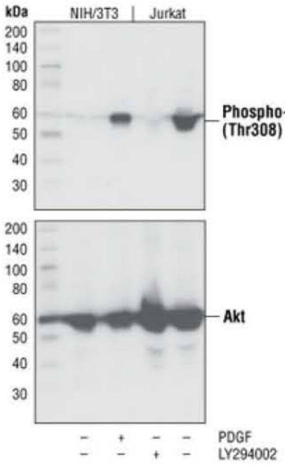

L. GSK3b (S9), CST#9323

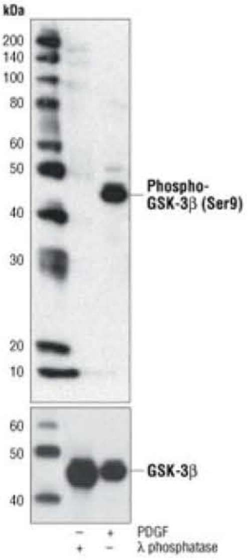

M. RelA (S536), CST#3033

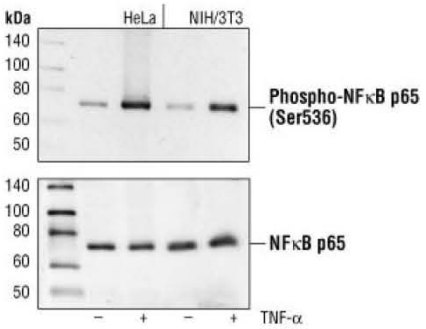

N. S6K1 (T389), CST#9205

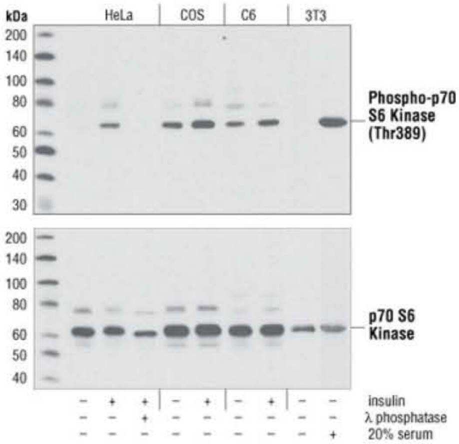

O. AMPKa (T172), CST#2535

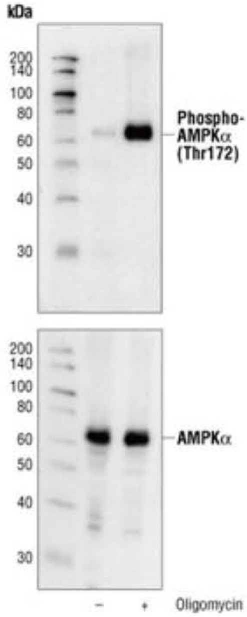

P. JNK1 (T183/Y185), CST#4668

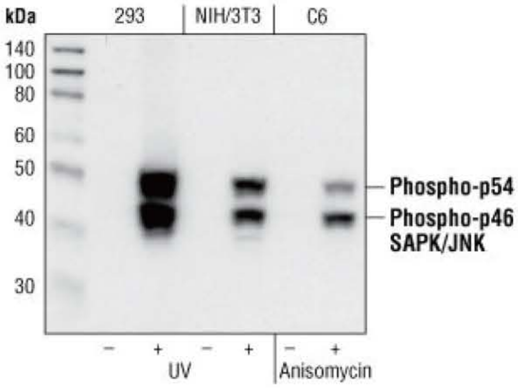

Q. VEGFR (Y951), CST#4991

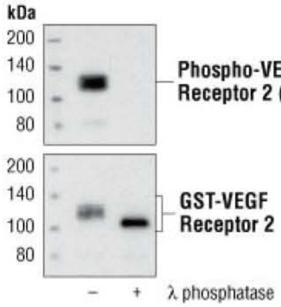

R. PKCδ/θ (S643/676), CST#9376

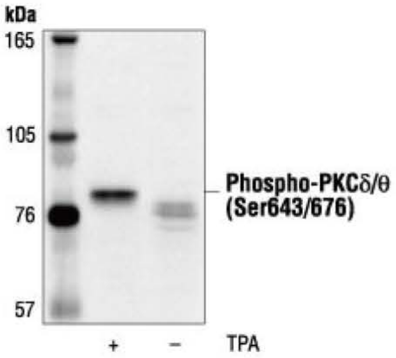

S. IR (Y1345), CST#3026

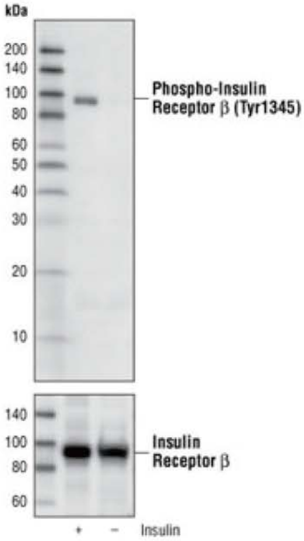

T. EGFR (Y1068), CST#3777

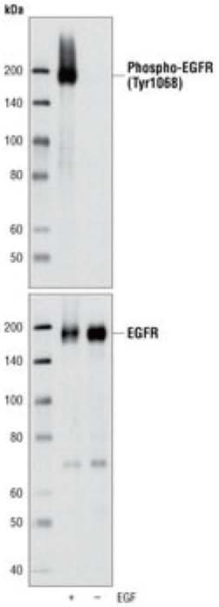

U. p53 (T81), CST#2676

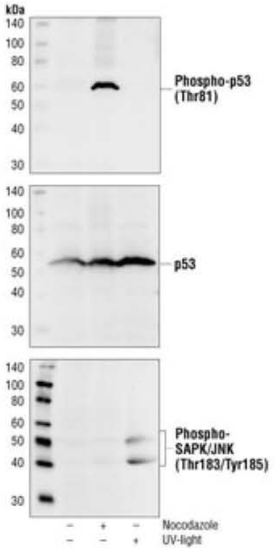

V. p53 (S37), CST#9289

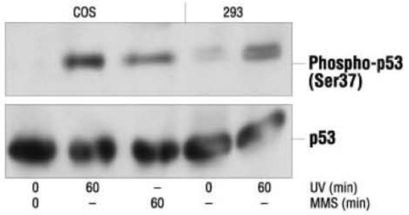

Supplement: Figure S2 — Phosphosite-specificity of the 22 antibodies on DPS. A–V. Western blot images to evaluate the phosphosite-specificity of the 22 antibodies in human samples collected from the Cell Signaling Technology. Western blotting data show that each antibody used detects specifically the changes in its target phosphorylation levels when the stimulation previously reported to alter the target phosphorylation is applied, compared to the antibody that recognizes the whole protein. For example, Fig. S2A shows that the Src(Y416)-specific antibody detect specifically the changes in the phosphorylation levels of Src at Y416 in serum starved COLO 201 cells when the cells are stimulated with 20% FBS for 5 minutes (upper). By contrast, the antibody Ab #2110 that recognizes the whole Src protein does not detects the phosphorylation changes at Y416 (lower). Note that the positions of the phosphosites in Fig. S2 differ from the ones in the text because the phosphosite-specificity data shown here are obtained from human samples, while the phosphosites in this study are detected from mouse samples (Table S2). The experimental designs to evaluate the phosphosite-specificity by Western blotting for the 22 antibodies were summarized in Table S3. (PDF) [file pone.0096456.s002.pdf]
