# Supplementary material for: Phosphokinase Antibody Arrays on Dendron-Coated Surface
Source: PLoS One. 2014 May 6;9(5):e96456. doi: 10.1371/journal.pone.0096456 (PMC4011796; doi:10.1371/journal.pone.0096456)
Supplement: Table S1 — Characteristics of commercial phosphokinase antibody arrays. The information for five commercial phosphokinase antibody arrays is summarized. *The number of phosphosite-specific antibodies in each array is as of April 2013. (PDF) [file pone.0096456.s005.pdf]

**Table S1. Characteristics of commercial phosphokinase antibody arrays.** The information for five commercial phosphokinase antibody arrays is summarized. *The number of phosphosite-specific antibodies in each array is as of April 2013.

| Company | Product Name | Coating Materials | Number of Phosphosites | Suggested Sample Amount | Detection Scheme |
|---|---|---|---|---|---|
| **Full Moon BioSystems** | **Phospho Explorer Antibody Microarray** | 3D polymer-coated glass slide | 675 phospho-specific Ab in a set of 1,170* Ab | 40-100 μg | Biotinylation and detection with Cy3-streptavidin |
| **Hypromatrix** | **Signal Transduction AntibodyArray** | Nitrocellulose membrane | A set of 400 Ab (no phospho-specific Ab) | Not mentioned | Detection with HRP-conjugated anti-phosphotyrosine Ab |
| **Kinexus Bioinformatics** | **Kinex Antibody Microarray Services** | No information available | ~340 phosphosite-specific Ab for at least 287 different phosphosites | 100 μg | Direct detection with a single Cy dye |
| **R&D Systems** | **Human Phospho-Kinase Array Kit** | Nitrocellulose membrane | 43 phospho-specific Ab in a set of 45 Ab | 200-600 μg cell lysate | Biotinylation and detection with HRP-streptavidin |
| **Sigma Aldrich** | **Panorama Ab Microarray - Cell Signaling Kit** | Nitrocellulose-coated glass slide | 27 phospho-specific Ab in a set of 224 Ab | 20-100 μg | Direct detection with Cy3 and Cy5 |
