# Supplementary material for: Phosphokinase Antibody Arrays on Dendron-Coated Surface
Source: PLoS One. 2014 May 6;9(5):e96456. doi: 10.1371/journal.pone.0096456 (PMC4011796; doi:10.1371/journal.pone.0096456)
Supplement: Table S2 — Properties of antibodies spotted on the DPA. The information for the 22 phosphosite-specific antibodies is summarized. All the 22 antibodies (catalog number: CST#) were purchased from Cell Signaling Technology. The residues of S, T, and Y represent serine, threonine, and tyrosine, respectively. The number after the residue represents the residue position in the corresponding protein. Phosphosite(H,M) indicates the position of the phosphorylation residue in human (H) and mouse (M) based on the PhosphositePlus database [45]. The pathways indicate the ones in which the corresponding protein is involved. ‘Host/Clonality’ represents the hosts from which mono- or poly-antibodies were generated. The reactivity of H or M indicates whether the antibodies are active in human (H) and mouse (M). (PDF) [file pone.0096456.s006.pdf]

Table S2. Properties of antibodies spotted on the DPA. The information for the 22 phosphosite-specific antibodies is summarized. All the 22 antibodies (catalog number: CST#) were purchased from Cell Signaling Technology. The residues of S, T, and Y represent serine, threonine, and tyrosine, respectively. The number after the residue represents the residue position in the corresponding protein. Phosphosite(H,M) indicates the position of the phosphorylation residue in human (H) and mouse (M) based on the PhosphositePlus database 39. The pathways indicate the ones in which the corresponding protein is involved. 'Host/Clonality' represents the hosts from which mono- or poly-antibodies were generated. The reactivity of H or M indicates whether the antibodies are active in human (H) and mouse (M).

Protein	Phosphosite (H, M)	Kinase	Pathway	Host/Clonality	Reactivity (H, M)	CST#
Src	Y416, Y418	Auto	Focal adhesion	Rabbit Mono	H, M	2113
CREB1	S133, S133	PKA, p90RSK, MSK, CaMKIV, MK2	cAMP	Rabbit Mono	H, M	9198
PLCγ	Y783, Y783	RTK, Syk	Calcium signaling	Rabbit Poly	H, M	2821
STAT3	Y705, Y705	JAK, FGFR, Src	Jak-STAT	Rabbit Mono	H, M	9145
STAT5a/b	Y694/Y699, Y694/Y699	JAK	Jak-STAT	Rabbit Mono	H, M	4322
PDGFR	Y751, Y750	PDGFR (auto)	PDGF	Mouse Mono	H, M	3166
YBX1	S102, S100	AKT, RSK	RNA processing	Rabbit Mono	H, M	2900
ERK1/2	T202/Y204, T203/Y205	MEK	MAPK	Rabbit Mono	H, M	4377
P38	T180/Y182, T180/Y182	RET, MAPK6	MAPK	Rabbit Mono	H, M	4511
Akt1	S473, S473	mTORC2	PI3K-Akt	Rabbit Mono	H, M	4058
Akt1	T308, T308	PDK1	PI3K-Akt	Rabbit Mono	H, M	2965
GSK3b	S9, S9	Akt	Wnt/β-Catenin	Rabbit Mono	H, M	9323
RelA	S536, S534	IKKa	NF-κB	Rabbit Mono	H, M	3033
S6K1	T389, T390	mTORC1	mTOR	Rabbit Poly	H, M	9205
AMPKa	T172, T172	LKB1	mTOR/PI3K-Akt	Rabbit Mono	H, M	2535
JNK1	T183/Y185, T183/Y185	MAP2K7, MAP2K4	MAPK	Rabbit Mono	H, M	4668
VEGFR	Y951, Y949	VEGFR (auto)	VEGF	Rabbit Mono	H, M	4991
PKCδ/θ	S643/676, S643/676	PKCd (auto)	Calcium signaling	Rabbit Poly	H, M	9376
IR	Y1345	IR (auto)	Insulin signaling	Rabbit Mono	H	3026
EGFR	Y1068, Y1069	EGFR (auto)	EGF	Rabbit Mono	H, M	3777
p53	T81	ATM, ERK, p38	P53, Cell cycle	Rabbit Poly	H	2676
p53	S37	ATM, ERK, p38	P53, Cell cycle	Rabbit Poly	H	9289
