# Supplementary material for: Phosphokinase Antibody Arrays on Dendron-Coated Surface
Source: PLoS One. 2014 May 6;9(5):e96456. doi: 10.1371/journal.pone.0096456 (PMC4011796; doi:10.1371/journal.pone.0096456)
Supplement: Table S3 — Phosphosite-specificity information of the 22 antibodies on DPA. Experimental design for Western blotting to evaluate the phosphosite-specificity of each antibody were summarized from the documents provided by Cell signaling Technology. (PDF) [file pone.0096456.s007.pdf]

Table S3. Phosphosite-specificity information of the 22 antibodies on DPA. Experimental design for Western blotting to evaluate the phosphosite-specificity of each antibody were summarized from the documents provided by Cell signaling Technology.

Protein	Phosphosite (H, M)	CST#	Specificity	Western Blotting
Src	Y416, Y418	2113	Phospho-Src Family (Tyr416) (100F9) Rabbit mAb detects endogenous levels of Src only when phosphorylated at tyrosine 416. The antibody may cross-react with other Src family members (Lyn, Fyn, Lck, Yes and Hck) when phosphorylated at equivalent sites.	Western blot analysis of Colo201 serum starved cells and stimulated with 20% FBS for 5 min, using Phospho-Src family (Tyr416) (100F2) Rabbit mAb (upper) and Src (L4A1) Mouse mAb #2110 (lower). (Fig S2A)
CREB1	S133, S133	9198	Phospho-CREB (Ser133) (87G3) Rabbit mAb detects endogenous levels of CREB only when phosphorylated at serine 133. The antibody also detects the phosphorylated form of the CREB-related protein, ATF-1.	Western blot analysis of extractsfrom SK-N-MC cells, untreated or forskolin- and FGF-treated, using Phospho-CREB (Ser133) (87G3) Rabbit mAb (upper) or CREB (48H2) Rabbit mAb #9197 (lower). (Fig S2B)
PLCγ	Y783, Y783	2821	Polyclonal antibodies are produced by immunizing animals with a synthetic phosphopeptide corresponding to residues surrounding tyrosine 783 of human PLCγ1. Antibodies are purified by protein A and peptide affinity chromatography.	Western blot analysis of extracts from NIH/3T3 cells, untreated or PDGF-stimulated for the indicated times, using Phospho-PLCγ1 (Tyr783) Antibody (upper) or PLCγ1 Antibody #2822 (lower). (Fig S2C)
STAT3	Y705, Y705	9145	Phospho-Stat3 (Tyr705) (D3A7) XP® Rabbit mAb detects endogenous levels of Stat3 only when phosphorylated at tyrosine 705. This antibody does not cross-react with phospho-EGFR or the corresponding phospho-tyrosines of other Stat proteins.	Western blot analysis of extracts from IFN-alpha treated Jurkat cellsand HeLa cells (left), as well as EGF treated A431 cells (right), using Phospho-Stat3 (Tyr705) (D3A7) XP®Rabbit mAb. Note that the basal phospho-Stat3 in A431 is detected by the antibody. (Fig S2D)
STAT5a/b	Y694/Y699, Y694/Y699	4322	Phospho-Stat5 (Tyr694) (D47E7) XPTM Rabbit mAb detects endogenous levels of Stat5a only when phosphorylated at Tyr694 and Stat5b when phosphorylated at Tyr699.	Western blot analysis of extracts from UT-7 cells, untreated or treated with erythropoietin (EPO; 3 units/ml for 5 min), TF-1 cells, untreated or treated with Human Granulocyte Macrophage Colony Stimulating Factor #8922 (hGM-CSF; 100ng/ml for 10 min), and NK-92 cells, untreated or treated with Human Interleukin-2 #8907 (hIL-2; 100ng/ml for 10 min), using Phospho-Stat5 (Tyr694) (D47E7) XP® Rabbit mAb (upper) or totalStat5 (3H7) Rabbit mAb #9358 (lower). (Fig S2E)
PDGFR	Y751, Y750	3166	Phospho-PDGF Receptor β (Tyr751) (88H8) Mouse mAb detects endogenous levels of PDGF receptor β only when phosphorylated at tyrosine 751.The antibody may cross-react with PDGF receptor α when highly overexpressed.	Western blot analysis of extracts from NIH/3T3 cells, untreated or treated with PDGF (50 ng/ml for 5 minutes) and/or calf intestinal phosphatase (CIP) as indicated, using Phospho-PDGF Receptor β (Tyr751) (88H8) Mouse mAb. (Fig S2F)
YBX1	S102, S100	2900	Phospho-YB1 (Ser102) (C34A2) Rabbit mAb detects endogenous levels of YB1 protein only when phosphorylated on Ser102.	Western blot analysis of extracts from MCF-7 cells, serum-starvedovernight and then either left untreated or treated with IGF-1 (50 ng/ml) for one hour, using Phospho-YB1 (Ser102) (C34A2) Rabbit mAb (upper) or YB1 Antibody #2749 (lower). Further treatment of the IGF-1-treated cell extracts with λ phosphatase depleted the phospho-specific YB1 signal (upper), but not total YB1 (lower). (Fig S2G)
ERK1/2	T202/Y204, T203/Y205	4377	Phospho-p44/42 MAPK (Erk1/2) (Thr202/Tyr204) (197G2) Rabbit mAb detects endogenous levels of p44 and p42 MAP Kinase(Erk1 and Erk2) when dually phosphorylated at Thr202 and Tyr204 of Erk1 (Thr185 and Tyr187 of Erk2), and singly phosphorylated at Tyr204. The antibody does not cross-react with the corresponding phosphorylated residues of either JNK/SAPK or p38 MAP kinase.	Western blot analysis of purifiedMAPK phospho-proteins or extracts from NIH/3T3 cells treated with UV light and PDGF, using Phospho-p44/42 MAPK (Erk1/2) (Thr202/Tyr204) (197G2) Rabbit mAb (upper), Phospho-p38 MAPK (Thr180/Tyr182) (3D7) Rabbit mAb #9215 (middle), and Phospho-SAPK/JNK (Thr183/Tyr185) (98F2) Rabbit mAb #4671 (lower). (Fig S2H)
P38	T180/Y182, T180/Y182	4511	Phospho-p38 MAPK (Thr180/Tyr182) (D3F9) XP® Rabbit mAb detects endogenous levels of p38 MAPK only when phosphorylated at Thr180 and Tyr182. This antibody does not cross-react with the phosphorylated forms of either p42/44 MAPK or SAPK/JNK.	Western blot analysis of extracts from COS and 293 cells, untreated or UV-treated, using Phospho-p38 MAPK (Thr180/Tyr182) (D3F9) XP®Rabbit mAb (upper) or p38 MAPK Antibody #9212 (lower). (Fig S2I)
Akt1	S473, S473	4058	Phospho-Akt (Ser473) (193H12) Rabbit mAb detects endogenous levels of Akt only when phosphorylated at Ser473.	Western blot analysis of extracts from untreated or PDGF-treated NIH/3T3 cells, pretreated with wortmannin #9951 and/or rapamycin #9904 as indicated, using Phospho-Akt (Ser473) (193H12) Rabbit mAb (upper) or Akt Antibody #9272 (lower). (Fig S2J)
Akt1	T308, T308	2965	Phospho-Akt (Thr308) (C31E5E) Rabbit mAb detects endogenous levels of Akt only when phosphorylated at Thr308.	Western blot analysis of extracts from NIH/3T3 and Jurkat cells, untreated, PDGF-treated or LY294002-treated as indicated, using Phospho-Akt (Thr308) (C31E5E) Rabbit mAb (upper) or Akt (pan) (C67E7) Rabbit mAb #4691 (lower). (Fig S2K)
GSK3b	S9, S9	9323	Phospho-GSK-3β (Ser9) (5B3) Rabbit mAb detects endogenous levels of GSK-3β only when phosphorylated at Ser9. The antibody may cross-react weakly with the phosphorylated form of GSK-3α due to high sequence homology.	Western blot analysis of extractsfrom NIH/3T3 cells, λ-phosphatase- or PDGF-treated, using Phospho-GSK-3β (Ser9) (5B3) Rabbit mAb (upper) or GSK-3β (27C10) Rabbit mAb #9315 (lower). (Fig S2L)

RelA	S536, S534	3033	Phospho-NF-kappaB p65 (Ser536) (93H1) Rabbit mAb detects NF-κB p65 only when phosphorylated at Ser536. It does not cross-react with the p50 subunit or other related proteins.	Western blot analysis of extractsfrom HeLa and NIH/3T3 cells, untreated or TNF-α treated (#2169, 20 ng/ml for 5 minutes), using Phospho-NF-κB p65 (Ser536) (93H1) Rabbit mAb (upper) or NF-κ B p65 Antibody #3034 (lower). (Fig S2M)
S6K1	T389, T390	9205	Phospho-p70 S6 Kinase (Thr389) Antibody detects endogenous levels of p70 S6 kinase only when phosphorylated at threonine 389. This antibody also detects p85 S6 kinase when phosphorylated at the analogous site (Thr412), and possibly S6KII phosphorylated at Thr401.	Western blot analysis of HeLa, COS, C6 and 3T3 cells, serum-starvedovernight, then treated with insulin, lambda-phosphatase or 20% serum as indicated. Upper panel probed with Phospho-p70 S6 Kinase (Thr389) Antibody #9205; lower panel probed with p70 S6 Kinase Antibody #9202. (Fig S2N)
AMPKa	T172, T172	2535	Phospho-AMPKα (Thr172) (40H9) Rabbit mAb detects endogenous AMPKα only when phosphorylated at threonine 172. The antibody detects both α1 and α2 isoforms of the catalytic subunit, but does not detect the regulatory β or γ subunits.	Western blot analysis of extracts from C2C12 cells, untreated or oligomycin-treated (0.5 μM), using Phospho-AMPKα (Thr172) (40H9) Rabbit mAb (upper) or AMPKα Antibody #2532 (lower). (Fig S2O)
JNK1	T183/Y185, T183/Y185	4668	Phospho-SAPK/JNK (Thr183/Tyr185) (81E11) Rabbit mAb detects endogenous levels of p46 and p54 SAPK/JNK only when phosphorylated at Thr183 and Tyr185. This antibody may cross-react with phosphorylated p44/42 or p38 MAP kinases.	Western blot analysis of extracts from 293 cells, untreated or UV-treated, NIH/3T3 cells, untreated or UV-treated and C6 cells, untreated or anisomycin-treated, using Phospho-SAPK/JNK (Thr183/Tyr185) (81E11) Rabbit mAb. (Fig S2P)
VEGFR	Y951, Y949	4991	Phospho-VEGF Receptor 2 (Tyr951) (15D2) Rabbit Monoclonal Antibody detects endogenous levels of VEGF receptor 2 only when phosphorylated at Tyr951. The antibody may slightly cross-react with activated VEGF receptor 1, but not with other related tyrosine phosphorylated tyrosine kinases.	Western blot analysis of recombinant human GST-VEGF Receptor 2 (Val789-Val1356), untreated or λ phosphatase-treated, using Phospho-VEGF Receptor 2 (Tyr951) (15D2) Rabbit mAb (upper) and VEGF Receptor 2 Antibody # 2472 (lower). (Fig S2Q)
PKCδ/θ	S643/676, S643/676	9376	Phospho-PKCdelta/theta (Ser643/676) Antibody detects endogenous levels of PKCdelta only when phosphorylated at serine 643, and PKCtheta only when phosphorylated at serine 676. This antibody does not cross-react with the phosphorylated PKC isoforms alpha, beta, gamma or epsilon.	Western blot analysis of extracts from U-937 cells, untreated or TPA-treated (0.2 μM), using Phospho-PKCdelta/theta (Ser643/676)Antibody. (Fig S2R)
IR	Y1345	3026	Phospho-Insulin Receptor (Tyr1345) (14A4) Rabbit mAb detects transfected levels of insulin receptor β only when phosphorylated at Tyr1345. The antibody slightly cross-reacts with activated IGF-I receptors and some other activated tyrosine kinases.	Western blot analysis of CHO IR/IRS-1 cells overexpressing human insulin receptor, untreated or treated with insulin (100 nM for 5 min), using Phospho-Insulin Receptor β (Tyr1345) (14A4) Rabbit mAb (upper) or Insulin Receptor β (4B8) Rabbit mAb #3025 (lower). (Fig S2S)
EGFR	Y1068, Y1069	3777	Phospho-EGF Receptor (Tyr1068) (D7A5) XP® Rabbit mAb detects endogenous EGF receptor only when phosphorylated at Tyr1068. This antibody may cross-react weakly with other tyrosine-phosphorylated proteins.	Western blot analysis of extracts of BxPC-3 cells, untreated or EGF-stimulated, using Phospho-EGF Receptor (Tyr1068) (D7A5) XP®Rabbit mAb (upper) and EGFReceptor Antibody #2232 (lower). (Fig S2T)
p53	T81	2676	Phospho-p53 (Thr81) Antibody detects endogenous levels of p53 only when phosphorylated at threonine 81.	Western blot analysis of extracts from HT29 cells, untreated, nocodazole-treated (50 ng/ml, 24h) or UV-treated (50mJ/cm2, 1hr), using Phospho-p53 (Thr81) Antibody (upper), p53 (1C12) Mouse mAb #2524 (middle), or Phospho-SAPK/JNK(T183/Y185) (98F2) Rabbit mAb #4671 (lower). (Fig S2U)
p53	S37	9289	Phospho-p53 (Ser37) Antibody detects endogenous levels of p53 only when phosphorylated at serine 37. It does not cross-react with p53 phosphorylated at other sites.	Western blot analysis of extracts from COS cells treated with UV or MMS, and 293 cells treated with UV, using Phospho-p53 (Ser37) Antibody (upper) or p53 Antibody #9282 (lower). (Fig S2V)

These specificity information in **Table S3** and **Fig. S2.** were provided by Cell Signaling Technology.
