# Supplementary material for: Phosphokinase Antibody Arrays on Dendron-Coated Surface
Source: PLoS One. 2014 May 6;9(5):e96456. doi: 10.1371/journal.pone.0096456 (PMC4011796; doi:10.1371/journal.pone.0096456)
Supplement: Table S4 — Percentage CVs of signal intensities for the 22 phosphosites in individual samples and standard deviations (SD) of the 22 phosphosites. In each sample, the intensities of four technical replicates for each phosphosite were used to compute the percentage CV. For each phosphosite, the pooled standard deviation was computed using the data of the three biological replicates each of which has the four technical replicates. (PDF) [file pone.0096456.s008.pdf]

Table S4. Percentage CVs of signal intensities for the 22 phosphosites in individual samples and standard deviations (SD) of the 22 phosphosites. In each sample, the intensities of four technical replicates for each phosphosite were used to compute the percentage CV. For each phosphosite, the pooled standard deviation was computed using the data of the three biological replicates each of which has the four technical replicates.

		2month								6month							
Proteins	phosphosite (H, M)	CV(%)			SD of	CV(%)			SD of	CV(%)			SD of	CV(%)			SD of
		Control1	Control2	Control3	Control1-3	AD1	AD2	AD3	AD1-3	Control1	Control2	Control3	Control1-3	AD1	AD2	AD3	AD1-3
Src	Y416, Y418	8.11	4.88	3.42	1350.30	6.39	2.15	3.69	1038.76	12.22	3.15	3.10	2424.01	1.00	4.29	5.41	742.28
CREB1	S133, S133	15.32	0.80	6.60	2098.30	12.30	20.74	0.87	2854.72	3.77	2.80	3.50	766.35	13.05	10.65	6.79	1847.16
PLCγ	Y783, Y783	11.08	4.82	3.17	1574.03	1.17	8.15	2.52	1087.19	4.30	0.91	2.12	673.96	7.08	3.21	4.68	1059.05
STAT3	Y705, Y705	18.05	4.46	2.85	1776.17	2.61	5.66	4.46	993.50	2.72	0.81	11.18	1001.97	2.93	1.51	6.40	1023.72
STAT5a/b	Y694/Y699, Y694/Y699	11.09	4.92	5.48	3340.76	3.73	5.82	4.44	2354.88	18.16	9.06	4.87	5460.61	2.74	4.36	2.83	1217.51
PDGFR	Y751, Y750	0.60	10.52	10.18	3540.40	10.57	2.65	5.71	3551.51	9.66	8.95	4.78	2938.71	1.39	2.96	4.95	765.71
YBX1	S102, S100	7.92	1.25	1.00	1148.82	12.42	3.90	18.20	2669.52	2.78	2.63	1.83	527.21	2.44	3.52	10.66	1324.31
ERK1/2	T202/Y204, T203/Y205	3.85	4.57	4.63	1036.97	5.14	9.48	1.43	1279.00	9.90	7.86	7.32	1790.98	2.24	1.75	6.03	1104.88
P38(MAPK14)	T180/Y182, T180/Y182	1.70	6.48	3.82	1494.07	5.48	7.02	2.48	1622.93	1.05	1.61	6.16	1043.85	2.57	1.85	8.76	1265.47
Akt1	S473, S473	2.79	2.56	6.98	969.06	2.52	3.89	4.83	745.66	6.84	3.40	8.81	1633.95	1.48	4.81	5.25	686.22
Akt1	T308, T308	6.16	9.10	4.19	2845.95	8.88	1.91	6.36	2457.63	11.64	6.46	1.65	2350.52	6.73	3.45	7.35	1480.79
GSK3b	S9, S9	4.39	1.76	4.11	431.39	3.96	6.22	5.00	569.29	3.52	0.90	2.88	512.08	2.56	2.71	4.19	433.12
RelA(p65)	S536, S534	14.07	7.42	2.90	1767.77	4.73	2.41	3.75	791.32	2.54	4.95	1.03	548.87	3.90	1.71	15.97	1713.12
S6K1	T389, T390	7.01	1.01	3.99	1091.27	4.79	4.44	4.17	1101.62	1.96	2.08	6.25	869.93	3.17	1.68	5.78	879.39
AMPKa	T172, T172	5.66	1.65	6.07	1045.22	16.25	13.48	0.63	2372.70	5.82	2.80	7.86	1058.96	0.61	6.39	8.02	1574.04
JNK1(MAPK8)	T183/Y185, T183/Y185	7.79	20.56	13.84	2639.55	15.02	5.06	11.39	1643.10	12.38	11.98	6.41	2355.47	5.20	13.50	8.66	1303.62
VEGFR	Y951, Y949	5.60	8.40	2.57	1806.01	13.27	0.80	9.23	2940.75	4.44	10.02	3.38	2008.00	4.49	7.22	6.26	1552.49
PKCδ/θ	S643/676, S643/676	5.59	9.45	8.49	1603.82	7.93	5.62	4.49	1040.08	9.67	9.17	4.87	1716.26	4.63	7.49	3.14	986.64
IR	Y1345	13.60	15.86	11.73	2407.47	4.56	3.22	5.28	1088.16	17.30	8.39	4.41	1925.82	3.00	4.70	8.24	1505.97
EGFR	Y1068, Y1069	1.92	4.85	0.38	926.13	2.37	6.06	4.67	1085.93	2.36	5.49	10.94	1741.64	3.81	3.03	6.82	1066.13
p53	T81	1.23	6.36	10.11	1565.35	10.13	4.83	7.41	1804.50	7.70	13.17	4.50	1799.19	3.34	5.12	1.77	862.49
p53	S37	4.25	3.06	3.05	702.46	4.07	9.69	3.06	1146.64	3.71	5.40	1.69	826.28	4.26	1.63	3.32	630.33
