# Supplementary material for: Phosphokinase Antibody Arrays on Dendron-Coated Surface
Source: PLoS One. 2014 May 6;9(5):e96456. doi: 10.1371/journal.pone.0096456 (PMC4011796; doi:10.1371/journal.pone.0096456)
Supplement: Table S5 — Statistical significance of the nine selected DPSs. For each of the nine DPSs, the direction of phosphorylation changes (U: up-regulated, D: down-regulated in AD compared to control), p-value from the post-hoc test with Bonferroni correction after ANOVA test, p-value from the median fold-change test, and log2-fold-change at two and six months are shown. (PDF) [file pone.0096456.s009.pdf]

Table S5. Statistical significance of the nine selected DPSs. For each of the nine DPSs, the direction of phosphorylation changes (U: up-regulated, D : down-regulated in AD compared to control), p-value from the post-hoc test with Bonferroni correction after ANOVA test, p-value from the median fold-change test, and log2-fold-change at two and six months are shown.

Proteins	phosphosite (H, M)	Direction		ANOVA test p-value		median fold test p-value		log_2-fold-change	
		2month	6month	2month	6month	2month	6month	2month	6month
PKCδ/θ	S643/676, S643/676	D	-	<0.001	-	0.05	0.23	-0.27	-0.21
EGFR	Y1068, Y1069	D	-	<0.001	-	0.02	1.00	-0.34	0.00
STAT3	Y705, Y705	U	U	<0.001	<0.001	0.04	0.01	0.27	0.59
RelA(p65)	S536, S534	-	U	-	<0.001	0.60	0.05	-0.05	0.41
Src	Y416, Y418	-	D	-	<0.001	0.10	0.00	0.21	-0.85
STAT5a/b	Y694/Y699, Y694/Y699	-	D	-	<0.01	0.13	0.02	0.19	-0.47
Akt1	S473, S473	-	D	-	<0.001	0.89	0.02	0.01	-0.52
GSK3b	S9, S9	-	D	-	<0.001	0.35	0.00	0.10	-1.05
JNK1(MAPK8)	T183/Y185, T183/Y185	-	D	-	<0.001	0.04	0.00	-0.28	-0.67
